# Dysferlin stabilizes membrane nanodomains of cardiomyocytes after myocardial infarction

**DOI:** 10.1038/s41598-026-42800-9

**Published:** 2026-03-26

**Authors:** Justus B. Wegener, Yannik Zühlke, Carolin Fleischhacker, Justus Marks, Brian Foo, Niklas Bader, Gabriel C. Riedemann, Jasper Wedemeyer, Kim-Chi Vu, Ana M. Vergel Leon, Nora Josefine Paulke, Tobias Kohl, Henning Urlaub, Constanze Schmidt, Gerd Hasenfuß, Tobias Moser, Eva A. Rog-Zielinska, Christof Lenz, Stephan E. Lehnart, Sören Brandenburg

**Affiliations:** 1 Department of Cardiology and Pneumology, University Medical Center Göttingen, Robert-Koch-Straße 42a, 37075 Göttingen, Germany; 2 Cellular Biophysics and Translational Cardiology Section, Heart Research Center Göttingen, University Medical Center Göttingen, Göttingen, Germany; 3 Cluster of Excellence “Multiscale Bioimaging from Molecular Machines to Networks of Excitable Cells” (MBExC), University of Göttingen, Göttingen, Germany; 4 DZHK (German Centre for Cardiovascular Research), Partner Site Göttingen, Göttingen, Germany; 5 Institute for Auditory Neuroscience and InnerEarLab, University Medical Center Göttingen, Göttingen, Germany; 6 Auditory Neuroscience and Synaptic Nanophysiology Group, Max Planck Institute for Multidisciplinary Science, Göttingen, Germany; 7 Department of Clinical Chemistry, University Medical Center Göttingen, Göttingen, Germany; 8 Institute for Experimental Cardiovascular Medicine, Faculty of Medicine, University Heart Center Freiburg-Bad Krozingen, University of Freiburg, Freiburg, Germany; 9 Bioanalytical Mass Spectrometry Group, Max Planck Institute for Multidisciplinary Sciences, Göttingen, Germany

**Keywords:** Dysferlin, Cardiomyocytes, Membrane repair, Myocardial infarction, Proteomics, Cardiology, Cell biology

## Abstract

Despite advances in acute care medicine, myocardial infarction (MI) remains a predominant cause of premature death and heart failure. In the MI border zone, cardiomyocytes are exposed to high biomechanical stress that impairs the integrity of the sarcolemmal membrane. Hence, we hypothesized that the Ca^2+^-sensitive membrane repair protein dysferlin is crucial for preserving sarcolemmal nanodomains in the MI border zone, like the transverse-axial tubule (TAT) network and the intercalated disc (ICD) membrane folds, and thereby limits the post-MI loss of myocardial function. We employed left anterior descending artery ligation to induce MI in wild-type (WT) versus dysferlin-knockout (KO) mice. While immunohistology identified an upregulated dysferlin expression of 230% in cardiomyocytes of the WT MI border zone, KO mice presented larger infarct sizes and reduced left-ventricular systolic function one week post-MI. To dissect the role of dysferlin in left-ventricular remodelling post-MI, we applied data-independent acquisition mass-spectrometry (DIA-MS) analysing the spatial proteomic profiles in WT versus KO hearts. In total, DIA-MS quantitated 5,700 proteins across all small samples, thereby identifying hundreds of genotype-specific proteomic changes for the left-ventricular infarct, border and remote zones one week post-MI. Complementing with our proteomic results, confocal and super-resolution stimulated emission depletion (STED) microscopy visualized severely degraded TAT membranes and enlarged ICD membrane folds in cardiomyocytes of the MI border zone. Importantly, extensive dysferlin signals clustered in vicinity to residual TAT structures and connexin-43 plaques at the ICD, indicating a stabilizing role of dysferlin in sarcolemmal nanodomain organization. In fact, co-immunoprecipitation-based DIA-MS and complexome profiling decoding the functional cardiac interactome of dysferlin confirmed prominent dysferlin interaction partners at TAT and ICD nanodomains. In conclusion, dysferlin represents a new molecular target that protects the integrity of sarcolemmal nanodomains in cardiomyocytes of the MI border zone, thereby reducing loss of contractility post-MI.

**Supplementary Information:**

The online version contains supplementary material available at 10.1038/s41598-026-42800-9.

## Introduction

In 2019, an estimated 5.8 million new cases of ischemic heart disease occurred in the 57 ESC member countries, which often first manifest as acute myocardial infarction (MI)^1^. While advances in the acute care of patients with MI have significantly reduced early mortality and event rates, effects on long-term survival have been less impressive^2^. Heart failure progression after MI depends not only on the extent of the immediate myocardial damage, but also on effective repair mechanisms in critically stressed cardiomyocytes. In the MI border zone, cardiomyocytes face hypoxia, inflammation and high biomechanical stress, all of which can compromise the integrity of the highly organized sarcolemmal membrane. Hence, we reason that membrane repair and remodelling mechanisms may be crucial to reduce the death of postmitotic cardiomyocytes in the MI border zone and to preserve contractility, ultimately lowering the burden of ischemic cardiomyopathy in patients.

The cardiomyocyte sarcolemma is a dynamic bilayer exposed to cyclical strain that provides a vital barrier, separating the cytosolic components from the extracellular environment, and integrates important cellular functions in specialized nanodomains. Regular invaginations of the outer surface sarcolemma form the transverse-axial tubule (TAT) endomembrane network, composed of transverse tubules (TT) at the Z-line of each sarcomere that are interconnected by axial tubule (AT) components^3,4^. Junctional membrane complexes of the TAT network with the sarcoplasmic reticulum (SR), known as cardiac dyads, mediate Ca^2+^-induced Ca^2+^ release in cardiomyocytes^5^, but undergo degradation in heart failure^6,7^. At the lateral cardiomyocyte cell–cell contact sites, the sarcolemma is highly folded at the intercalated discs (ICD) with electrical and mechanical junctions connecting individual cardiomyocytes in a functional syncytium^8^. The ICD electrical junctions or gap junctions are formed between neighbouring cells by two opposing connexons—pore-forming hemichannels each assembled of 6 connexin-43 proteins in ventricular cardiomyocytes^8^. Among various mechanical junctions of the ICD, we highlight the adherens junctions with the transmembrane protein N-cadherin, assembling the intercellular connection between cardiomyocytes and giving continuity to the contractile machinery of the cells^8^. However, little is known about the remodelling and plasticity of these functional membrane nanodomains in cardiomyocytes of the MI border zone, and about the local membrane fusion and repair mechanisms that may protect and shape them.

Dysferlin, a 237 kDa large Ca^2+^-binding transmembrane protein with 8 C_2_-domains^9^, facilitates Ca^2+^-dependent membrane fusion and repair events in striated muscle cells^10–13^. Loss-of-function mutations in *DYSF* cause a progressive phenotype of skeletal muscle disease referred to as limb girdle muscular dystrophy R2 (LGMD R2)^14^, associated with mild forms of dilated cardiomyopathy^15–17^. In skeletal muscle cells, dysferlin is thought to interact with other membrane-associated proteins like mitsugumin-53 (MG53)^18^ and the annexin protein family^19,20^, to recruit and fuse reparative vesicles to regions of damaged membrane. Importantly, hearts from dysferlin-knockout mice seem to exhibit an impaired recovery from ischemia/reperfusion injury^21–23^. Since dysferlin was recently found to localize in vicinity to TAT membranes and ICD membrane folds^24^, we hypothesized that dysferlin is crucial to stabilize these nanodomains in cardiomyocytes of the MI border zone, protecting the sarcolemmal integrity and ultimately reducing the loss of left-ventricular (LV) systolic function.

Here, we applied label-free data-independent acquisition mass-spectrometry (DIA-MS) to resolve the region-specific proteomic profiles each of the infarct zone, border zone and remote zone of wild-type (WT) versus dysferlin-knockout (KO) mouse hearts post-MI. Overall, DIA-MS quantitatively analysed 5,700 proteins, with significant proteomic profile changes between regions and genotypes. While dysferlin protein expression was upregulated in the MI border and remote zone of WT hearts, dysferlin deficiency highly influenced the region-specific proteomic remodelling post-MI, resulting in larger infarct size and loss of systolic LV function. Stimulated emission depletion (STED) nanoscopy in combination with electron tomography visualized dysferlin signals that accumulated in nanometric proximity to severely degraded TAT membranes and enlarged ICD membrane folds in the MI border zone, apparently stabilizing and repairing these sarcolemmal nanodomains in the face of high biomechanical stress. Finally, complementary techniques to study the cardiac dysferlin interactome suggested the ICD proteins connexin-43, N-cadherin and β-catenin as novel dysferlin interaction partners in functional high molecular weight complexes.

## Methods

### Myocardial infarction model

All animal procedures were conducted in accordance with the guidelines from Directive 2010/63/EU of the European Parliament on the protection of animals used for scientific purposes, and approved by the local veterinarian state authority (LAVES, Oldenburg, Germany; animal protocols no. T11.2 and 33.19-42502-04-21/3785). The study is reported in accordance with ARRIVE guidelines. Adult sex-mixed WT and dysferlin-KO^10^ mice in the C57BL/6J background for at least 10 generations, aged 8 to 16 weeks and at a bodyweight of 17.5–29 g, were used for MI vs sham surgery. After administration of intraperitoneal anaesthesia (medetomidine (0.5 mg/kg bodyweight), midazolam (5 mg/kg bodyweight), and fentanyl (0.05 mg/kg bodyweight)) and intubation of the trachea, the chest was opened via left thoracotomy, the pericardium removed, and a 7–0 polyamide suture with a U-shaped needle was passed under the proximal left anterior descending artery (LAD) in order to permanently occlude the vessel. Myocardial ischemia was confirmed by colour change of the anterior wall. No ligation was applied to sham-treated animals. 1- and 4-weeks after surgery, investigator-blinded transthoracic echocardiographic measurements were performed in the parasternal long and short axis views with ECG monitoring under 1% isoflurane anaesthesia.

### Mouse heart tissue collection

Mice were anesthetized with 2% isoflurane before cervical dislocation. Mouse hearts were collected 1- and 4-weeks after myocardial infarction and retrogradely perfused with 0.9% NaCl using a modified Langendorff-perfusion system at a flow rate of 4 mL/min for 2 min to wash out blood from the myocardium^26^. LV tissue for quantitative SDS-PAGE, DIA-MS, co-immunoprecipitation (coIP) proteomics and complexome profiling were dissected under a binocular zoom microscope.

### Human endomyocardial LV biopsies

LV biopsies were obtained from patients suffering from ischemic cardiomyopathy. Myocardial biopsies from non-failing (NF) donor hearts rejected for transplantation served as controls. The institutional ethics committee of the University Medical Center Göttingen approved the study (no. 10/5/16), the study conformed to the principles outlined in the Declaration of Helsinki, and written informed consent was obtained from all patients prior to the inclusion.

### Protein analysis

Quantitative SDS-PAGE was performed as described previously^24^. Twenty micrograms of protein per lane were resolved by SDS-PAGE on 4% to 20% Tris–HCl protein gradient gels. Full immunoblot scans are provided in Figure S1. For detailed information on primary antibodies, refer to Supplemental Table 1. Buffer compositions are outlined in Supplemental Table 2.

### Region-resolved tissue proteomics after myocardial infarction

After heart extraction and 2 min Langendorff-perfusion, myocardial tissue samples from the infarct zone, border zone and remote zone of infarcted mouse hearts were dissected under a binocular zoom microscope (Zeiss Stemi 305) using micro iris scissors and immediately snap frozen in liquid nitrogen. Following trypsination, myocardial tissue samples were lysed in 2% SDS, 100 mM HEPES buffer by pressure cycling technology^27^, tryptically digested using an SP3 protocol^28^ on amine-coated paramagnetic beads, and analysed by data-independent acquisition mass-spectrometry (DIA-MS) on a Bruker timsTOF Pro 2 mass-spectrometer. Detailed information is provided in the Supplementary Material.

### Cardiac dysferlin interactome

LV myocardium was prepared by ultracentrifugation (100,000 g) and solubilisation in buffer containing 0.15% CHAPS for coIP experiments. 500 µg of protein from WT and dysferlin-KO LV myocardium after membrane preparation were incubated with 4 µg anti-dysferlin antibody (ab124684) or unspecific rabbit IgG antibody. Consequently, Dynabeads Protein G (Thermo Fisher Scientific) were used to precipitate dysferlin and protein interaction partners. After three times washing and denaturation using SDS at 95 °C, the samples were separated by SDS-PAGE, digested in gel with trypsin, and analysed by DIA-MS on the Bruker timsTOF Pro 2.

### Complexome profiling

Membrane pellets (100,000 g) from LV myocardium were solubilized and fractionated based on apparent molecular mass by size-exclusion chromatography as described^29^. Each fraction was then subject to trypsin digestion followed by bottom-up mass-spectrometry based proteomic analysis (LC–MS/MS). Spectral data was analysed using Spectronaut v16.3. Reanalysis of published dataset to investigate co-elution of selected proteins of interest for this work was done on Microsoft Excel^29^.

### Confocal microscopy and STED nanoscopy

Mouse hearts and human endomyocardial biopsies were fixed in 4% PFA overnight, embedded in paraffin, and cut in 5 µm thick slices. Following deparaffinization and rehydration, epitopes were unmasked in sodium citrate buffer, and samples permeabilised and blocked for 1 h with 0.1% Triton-X100 and 4% bovine serum albumin. Histological slices were incubated with primary antibodies in blocking buffer overnight. For information on primary antibodies, refer to the Supplemental Table 1. Primary antibody specificity was verified by experiments including dysferlin-knockout mice (Figure S2). In addition, secondary antibody-only controls were used to distinguish genuine target staining from background. Finally, samples were incubated with secondary antibodies coupled to the fluorophores STAR635P and STAR580 (Abberior) for 2 h and mounted with ProLong Gold Antifade Mountant (Thermo Fisher Scientific). Confocal images were acquired with a Leica TCS SP8 laser-scanning microscope with HC PL APO C2S 100x/1.40 oil, HC PL APO CS2 40x/1.30 oil and HC PL FLUOTAR 10x/0.30 dry objectivse, while STED images were acquired with a HC-PL-APO-C2S 100x/1.40 oil objective. Images were processed in ImageJ/Fiji.

### Electron tomography

Mouse hearts were perfusion-fixed with isoosmotic Karnovsky’s fixative. Tissue fragments from the MI border zone were excised, washed with 100 mM sodium cacodylate, post-fixed in 1% OsO_4_, dehydrated in graded acetone, and embedded in Epon-Araldite resin. Semi‐thick sections (300 nm) were placed on formvar‐coated copper slot‐grids, post-stained with 2% aqueous uranyl acetate and Reynold’s lead citrate. Sections were imaged using 300 kV Tecnai TF30 (FEI Company, now Thermo-Fisher Scientific, Eindhoven, The Netherlands). Tilt series were aligned, reconstructed, and combined using IMOD as described previously^30^.

### Statistical analysis

Data are presented as mean ± SEM. *P* < 0.05 was considered indicative of statistical significance. Statistical analyses were performed using GraphPad Prism versions 9.4.0 or newer, IBM SPSS Statistics 29.0.0.0, and R version 4.3.1. Details regarding experimental design, sample sizes, normalization procedures, tests establishing normality, named statistical tests, post hoc correction for multiple comparisons, and precise *P* values are provided in the Supplemental Table: Statistical details, and the corresponding figure legends. Experiment-wide multiple test correction was not applied. Representative images were selected to visualize the average in each group.

## Results

### Increased dysferlin expression in the myocardial infarction border zone is associated with ameliorated loss of ventricular function post-MI

**Fig. 1:**
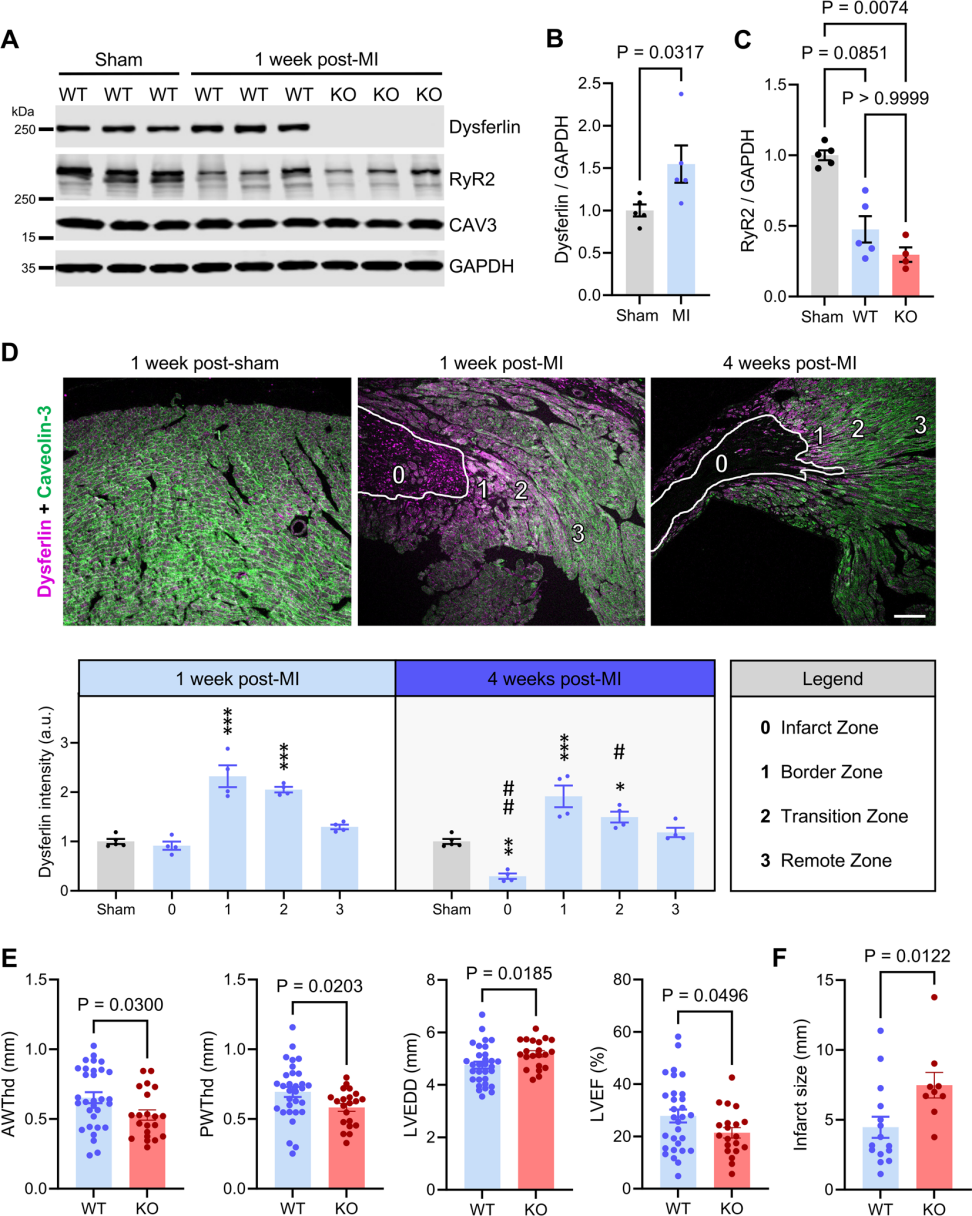
Dysferlin expression is highly increased at the MI border correlating with reduced loss of ventricular function post-MI. (**A** through **C**), Immunoblot of LV mouse heart lysates showing increased dysferlin and decreased ryanodine receptor type 2 (RyR2) protein expression 1-week post-MI. Infarct zones were excluded from experiments. CAV3, caveolin-3; GAPDH, glyceraldehyde 3-phosphate dehydrogenase. Immunoblot representative of 3 independent experiments. Images were cropped, original blots are presented in Figure S1. *n* = 5 WT sham, 5 WT MI vs 4 KO MI hearts. Mann–Whitney *U* test in (**B**), Kruskal–Wallis test in (**C**). (**D**) Immunohistology of dysferlin and the cardiomyocyte membrane marker caveolin-3 in WT LV tissue sections 1- and 4-weeks post-MI. Of note, dysferlin shows the highest expression level in the MI border zone (1); infarct zone (0) delineated in white. *n* = 5 1psham, 4 1pMI vs 4 4pMI hearts. 1-way ANOVA; * comparison to sham control, # comparison of 4pMI to 1pMI (*/# *P* < 0.05, **/## *P* < 0.01, ***/### *P* < 0.001). Scale bar 100 µm. (**E**) Transthoracic echocardiography comparing LV dimensions and systolic function of wild-type (WT) vs dysferlin-knockout (KO) mice 1-week after myocardial infarction (1pMI). AWThd, anterior wall thickness in diastole; LVEDD, left-ventricular enddiastolic diameter; LVEF; left-ventricular ejection fraction; PWThd, posterior wall thickness in diastole. *n* = 31 WT vs 21 KO mice. Unpaired Mann–Whitney *U* test (AWThd) and unpaired Welch *t* test (LVEDD, LVEF, PWThd). (**F**) KO mice develop significantly larger infarct sizes post-MI. *n* = 15 WT vs 9 KO mice. Unpaired Mann–Whitney *U* test.

In order to explore the role of the endogenous membrane repair protein dysferlin after MI, we employed permanent LAD ligation in WT versus dysferlin-KO mice at 8–16 weeks of age (see Methods and Supplemental Material for detailed information). Immunoblotting of WT LV tissue lysates 1-week post-MI (excluding the infarct zone) showed an increase in dysferlin protein expression to 155% compared to sham-treated myocardium (Fig. 1A,B; full-length blots are shown in Figure S1), which was similar between female and male mice. Notably, only full-length dysferlin and no cleavage products were observed in mouse heart tissues (Fig. 1A, Figure S1A)^18^. In contrast to the upregulation of dysferlin, we detected a decreased expression of the SR Ca^2+^ release channel ryanodine receptor type 2 (RyR2), which was recently established as a dysferlin protein interaction partner in cardiomyocytes, in both WT and KO myocardium post-MI (Fig. 1A,C)^24^.

To map the local expression of dysferlin in differential regions of the left ventricle and in the course of time post-MI, we performed immunohistology of dysferlin and caveolin-3 on LV tissue slices 1- and 4-weeks after surgery. While sham-treated LV myocardium showed a uniform distribution of dysferlin and caveolin-3 as expected, we identified a markedly increased dysferlin signal near the infarct zone (*magenta*), hereafter referred to as ‘border zone’ (Fig. 1D, top; see Figure S2 for the dysferlin-KO used as immunofluorescence specificity control). Quantitative analysis confirmed a 232% and 191% locally increased dysferlin signal intensity in the MI border zone 1- and 4-weeks post-MI, respectively (Fig. 1D, bottom). Since dysferlin expression gradually decreased with growing distance to the infarct zone, we additionally defined a ‘transition’ and ‘remote zone’ (Fig. 1D). The infarct zone presented no relevant caveolin-3 expression 1-week post-MI, but strong, abnormally condensed dysferlin signals, which were almost completely cleared 4-weeks post-surgery (Fig. 1D).

As local dysferlin upregulation may offer a biological benefit for hypoxically and mechanically stressed cardiomyocytes of the MI border zone, we sought to analyse the mortality and systolic LV function of dysferlin-KO animals post-MI. We anticipate that mortality as well as LV wall thickness and function were not changed between WT and dysferlin-KO mice prior to surgery (Figure S3)^24^. Only the LV enddiastolic diameter was slightly elevated in dysferlin mice at baseline (Figure S3). However, during an observation period of 25 days post-surgery, we noticed a trend toward higher mortality in dysferlin-KO versus WT animals (Figure S4A), which was particularly pronounced in male mice (Figure S4B-C). In addition, investigator-blinded transthoracic echocardiography 1-week post-MI revealed a decreased anterior and posterior wall thickness in dysferlin-KO versus WT mice (Fig. 1E, left; see Table S3 for detailed echocardiographic data). This may be explained by a reduced hypertrophic response of dysferlin-KO myocardium in LV volume-overload, as previously reported for LV pressure-overload^24^. Furthermore, left ventricles of dysferlin-KO animals were more dilated as demonstrated by the LV enddiastolic diameter, and presented a significantly decreased LV ejection fraction 1-week post-MI (LVEF 21% versus 28%, Fig. 1E, right). The difference in systolic LV function may partly be attributed to significantly larger infarct sizes in dysferlin-KO animals, as shown in Fig. 1F. Together, the protein expression of dysferlin is upregulated in LV cardiomyocytes of the MI border zone in WT mice, which correlates with a reduced infarct size and preserved LV function post-MI compared to KO mice.

### Dysferlin expression shapes proteomic profiles of the left ventricle post-MI

**Fig. 2:**
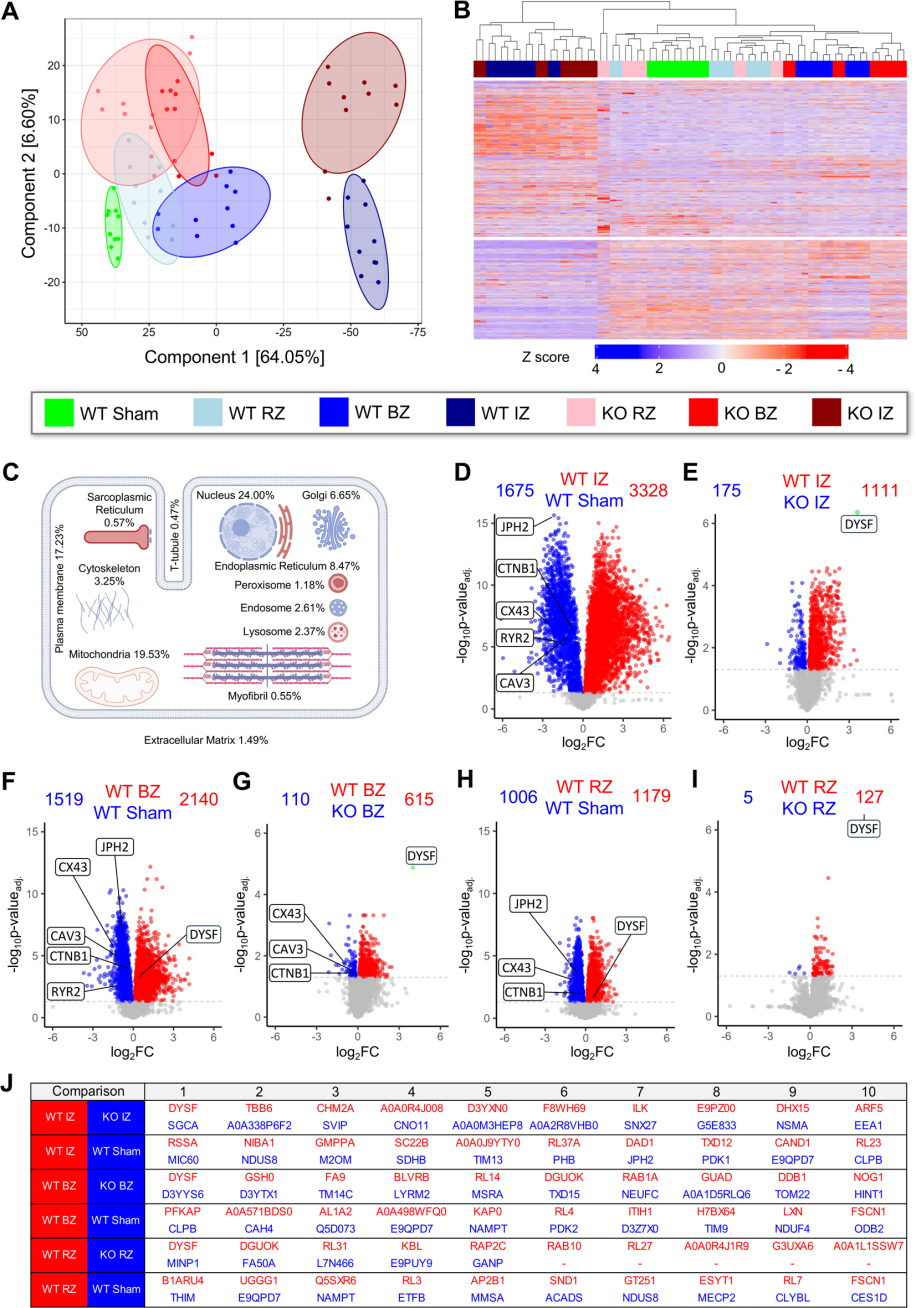
Dysferlin expression defines proteomic profiles of left-ventricular zones post-MI. Protein expression profiling of individual LV tissue fragments from the infarct zone (IZ), border zone (BZ) and remote zone (RZ) of WT and dysferlin-knockout (KO) mice 1-week post-MI vs sham-operated animals using label-free DIA-MS. *n* = 5 different mouse hearts independently analysed per group with two replicate injections per sample. 5,700 proteins were quantified, with 4,360 detected across all samples. (**A**) Principal component analysis projecting spatially resolved LV data onto component 1 and 2, with the percentage of total variance listed in parentheses (x/y axis legend). Ellipses indicate the 85% confidence intervals. Differential proteotype clusters can be segregated based on both MI zones and WT vs KO genotypes. (**B**) Heatmap with Euclidean clustering visualizes 5,602 differentially regulated proteins defined by blue-to-red colour table z score (ANOVA, FDR < 0.05). Legend box applies to (**A**,**B**). (**C**) VM cartoon demonstrates the percentages of the 5,602 significantly regulated proteins in the DIA-MS analysis mapped onto subcellular organelles as annotated in gene ontology cellular components. (**D** through **I**), Volcano plots with pairwise comparisons of WT MI zones vs sham LV myocardium or WT vs KO MI zones 1-week post-surgery. The number of significantly up- or downregulated proteins (*P* < 0.05) is indicated on top, and up- or down-regulated proteins are indicated by red or blue coloured dots, respectively. CAV3, caveolin-3; CTNB1, β-catenin; CX43, connexin-43; DYSF, dysferlin; JPH2, junctophilin-2; RYR2, ryanodine receptor type 2. J, Table summarizing the top ten up- or downregulated proteins in pairwise comparisons. All *P* values were adjusted for multiple testing using the Benjamini–Hochberg correction.

In order to dissect the local consequences of dysferlin deficiency for left-ventricular remodelling post-MI, we applied a previously established label-free DIA-MS protocol^7^ to analyse the region-specific proteomic profiles of the infarct, border and remote zone from WT and dysferlin-KO hearts, respectively. To optimally separate LV zones post-MI for DIA-MS analysis, we used a binocular zoom microscope and micro iris scissors to excise the border zone as a thin myocardial layer right next to the clearly demarked infarct zone. Based on *n* = 5 mouse hearts per genotype 1-week post-MI and 2 technical replicates per sample, we quantitatively analysed 5,700 proteins, with 4,360 detected across all samples (Fig. 2). Intriguingly, as visualized in the principal component analysis (PCA) plot (Fig. 2A), DIA-MS segregated proteomic profiles based on both LV zones post-MI on the one hand (horizontal axis, component 1; hereafter referred to as ‘proteotypes’) and WT versus KO genotypes on the other hand (vertical axis, component 2). The proteomic transition from sham-treated LV myocardium to remote zone > border zone > > > infarct zone post-MI is outlined along principal component 1 (PC1), reflecting more than 64% of the total difference between infarct zone proteotypes of both genotypes and the WT control proteotype (Fig. 2A). Furthermore, technical replicates grouped in close proximity, confirming the reproducibility of our protocol.

The heatmap of all 5,602 differentially abundant proteins highlights the robust difference of the infarct zone proteotype versus all other vital myocardial proteotypes (Fig. 2B), namely border and remote zone, and sham-treated LV tissue. The cardiomyocyte cartoon in Fig. 2C projects the percentages of differentially regulated proteins in these proteotypes onto subcellular organelles/compartments based on the gene ontology (GO) cellular component annotation. Most of the proteins were attributed to the nucleus, mitochondria, and the plasma membrane.

For more detailed analyses, pairwise region and genotype-specific comparisons are illustrated in Fig. 2D through I, and *Supplemental Data: Proteomics* provides full tables of differentially-regulated proteins with log_2_ fold changes and *P* values. While we highlighted the proteomic changes of the infarct zone (5,003 proteins), the border zone (3,659 proteins), and the remote zone (2,185 proteins) versus sham-treated myocardium (Fig. 2D,F,H), highly relevant changes also emerged in the direct WT versus dysferlin-KO comparisons (infarct zone 1,286, border zone 725, and remote zone 132 regulated proteins) (Fig. 2E,G,I). The high number of differentially abundant proteins in the direct genotype comparisons underlines the importance of the membrane repair protein dysferlin for LV remodelling post-MI. Fully in line with an upregulated dysferlin expression found by immunoblotting and immunohistology post-MI, dysferlin protein abundance was increased in the WT MI border and remote zones (Fig. 2F,H). In contrast, the SR and Ca^2+^-regulating proteins RyR2 and junctophilin-2 as well as the cardiomyocyte surface and TAT membrane protein caveolin-3^5,31^, previously recognized as dysferlin interacting proteins^24,32^, were decreased in the MI border zone compared to sham (Fig. 2F). Additionally, the ICD proteins β-catenin and connexin-43, the latter important for intercellular communication by forming the gap junction hemichannels at ICD cell–cell contact sites, were markedly decreased in the MI border zone (Fig. 2F). Finally, Fig. 2J summarizes the top 10 most up- and downregulated proteins in all pairwise comparisons.

In order to understand the underlying pathophysiological processes linked to the proteotype differences as outlined above, we applied Kyoto encyclopedia of genes and genomes (KEGG)^33,34^ pathway enrichment analysis using pathfindR (Figure S5). The Venn diagram in Figure S5A visualizes shared and specifically enriched KEGG pathways in the WT versus dysferlin-KO comparisons 1-week post-MI. Heatmaps of the top 50 enriched KEGG pathways per genotype can be found in Supplemental Figs. 6–8, and protein changes corresponding to individual KEGG pathways are summarized in *Supplemental Data: Proteomics*. For the WT versus dysferlin-KO comparisons of the infarct and border zone, we highlighted 5 enriched KEGG pathways, respectively (Figure S5B and C), and showed up- or downregulated proteins in term-gene graphs (Figure S5D and E). In the infarct zone comparison, dysferlin deficiency induced changes in proteins involved in apoptosis, hypoxia-inducible factor 1 (HIF-1) signalling, as well as B- and T-cell-mediated inflammatory response (Figure S5B), consistent with a previous report from Evans et al. that linked sustained inflammation to membrane repair incompetence in dysferlin-KO mice^23^. In the MI border zone comparison, dysferlin-KO-specific differences were related to adrenergic and Ca^2+^-signalling, gap junction organization, and HIF-1 signalling (Figure S5C). In summary, DIA-MS resolved the proteomic profiles of infarct, border and remote zones, and identified numerous proteomic changes in dysferlin-KO myocardium post-MI, which likely underlie pathophysiological processes that culminate in further loss of ventricular function post-MI.

### Transverse-axial tubule membranes are highly degraded in cardiomyocytes of the MI border zone

**Fig. 3:**
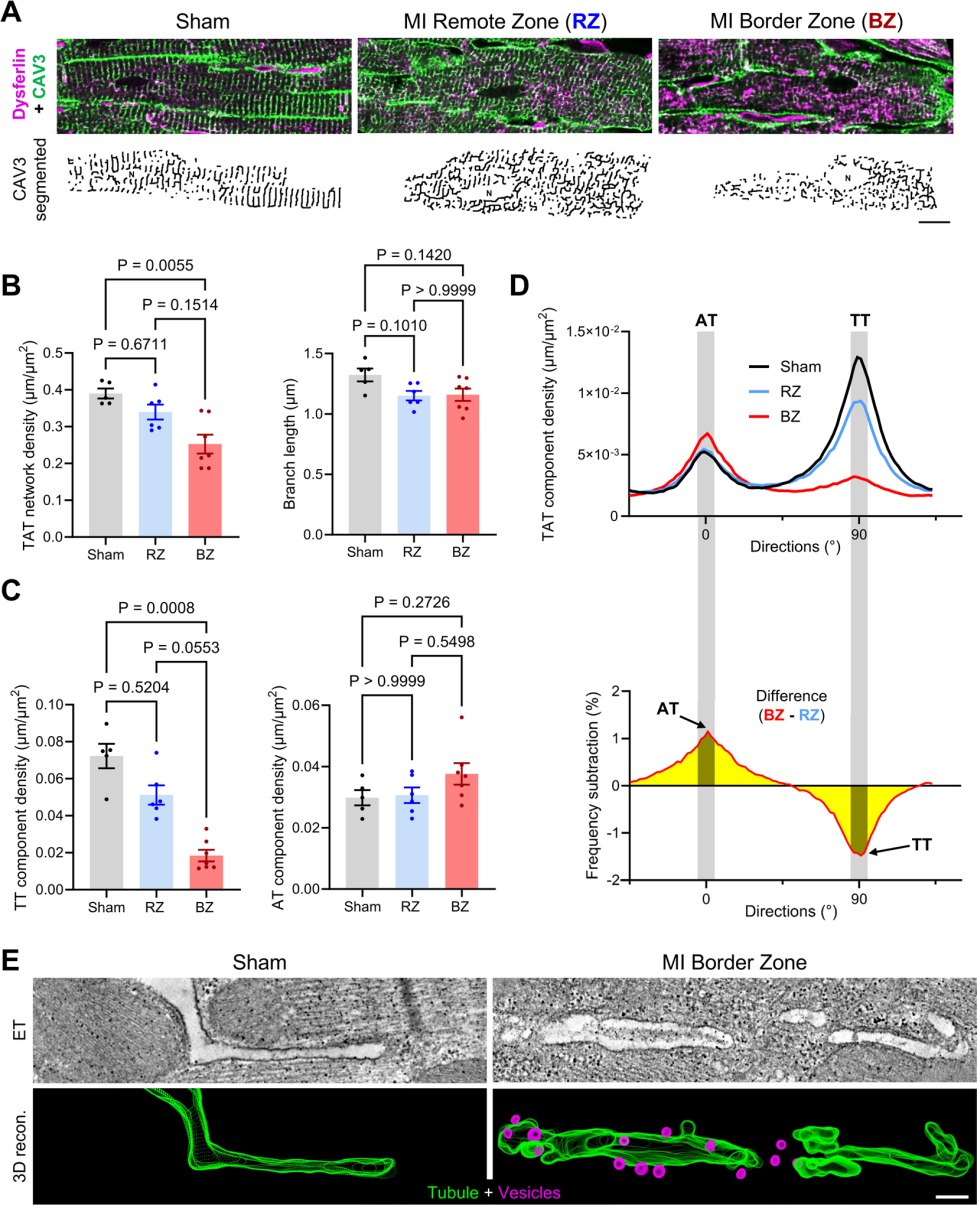
The transverse-axial tubule network is severely degraded in myocytes of the MI border zone. (**A**) *Top:* Confocal co-immunofluorescence imaging of dysferlin and the membrane and transverse-axial tubule (TAT) marker caveolin-3 (CAV3) in LV tissue slices of WT sham vs MI hearts 1-week post-surgery. The majority of the transverse tubule elements are lost in myocytes of the MI border zone (BZ), while residual (axial) tubules are tightly decorated with dysferlin signals (*magenta*). *Bottom:* CAV3 signals were subjected to segmentation protocols for detailed TAT network analysis in (**B**–**D**). Scale bar 10 µm. (**B**) The TAT network density is significantly reduced in myocytes of the MI border zone compared to sham, and the tubule branch length tends to be smaller in remote and border zone myocytes. Kruskal–Wallis test. (**C**) Directionality analysis reveals a substantial loss of transverse tubule (TT) components in BZ myocytes. No significant changes were observed for axial tubule (AT) elements of the TAT network in myocytes post-MI. Kruskal–Wallis test. (**D**) *Top:* Histogram shows the mean bimodal distribution of TAT network components in myocytes 1pMI: minor peak = AT (0°); major peak = TT (90°); binning ± 5° (grey columns). *Bottom:* Subtraction histogram (BZ minus RZ) highlights the increase of AT vs loss of TT components in myocytes of the MI border vs remote zone. *n* = 52/41/53 LV myocytes of 5 sham controls, 6 RZ vs 7 BZ from individual mouse hearts in (**B**–**D**). (**E**) Electron tomography (ET) and 3-dimensional reconstruction of representative volumes visualizing residual axial tubule components with lots of small membrane protrusions and intracellular vesicles in direct proximity to the TAT network in myocytes of the MI border zone. Scale bar 200 nm.

The membrane fusion and repair protein dysferlin was shown to be localized on tubular membranes and in a vesicular compartment next to tubular membranes^24^, continuous invaginations from the outer surface sarcolemma that are organized in a 3D TAT endomembrane network in cardiomyocytes^4^. Hence, we hypothesized that increased dysferlin expression post-MI may help to stabilize, repair and shape TAT membranes in order to preserve regulated excitation–contraction coupling. Notably, our region-resolved proteomic analysis revealed a decreased protein abundance of the TAT membrane protein caveolin-3 and the SR and Ca^2+^-regulating proteins RyR2 and junctophilin-2 in the MI border zone, the latter normally localized in the cardiac dyads (Fig. 2F). Confocal co-immunofluorescence imaging of dysferlin and caveolin-3 in LV tissue slices from WT mice was used to *a)* localize dysferlin subcellularly, and *b)* visualize and skeletonize the caveolin-3-positive TAT membrane network based on previously established protocols (Fig. 3A; see Figure S2 for the dysferlin-KO used as immunofluorescence specificity control)^7^. While LV cardiomyocytes in sham-treated WT myocardium showed a regular, well-organized TAT network mainly composed of TT components, TAT networks appeared disorganized in cardiomyocytes of the remote zone and were severely degraded in the border zone of WT myocardium 1-week post-MI (Fig. 3A, Figure S9). Interestingly, we observed slightly larger intracellular dysferlin signals next to TAT membranes of the remote zone (Fig. 3A). Membrane-decorating dysferlin signals were even larger and more intense in LV cardiomyocytes of the MI border zone (Fig. 3A).

Subsequently, caveolin-3 membrane skeletons were used for detailed TAT network analysis in LV cardiomyocytes post-MI (Fig. 3B–D; each measurement indicates an independent mouse heart). Compared to sham controls, the TAT network density significantly decreased in cardiomyocytes of the MI border zone (-35%), whereas the tubular branch length of individual components was not changed (Fig. 3B). In particular, transverse tubule (TT) components were highly degraded in cardiomyocytes of the MI border zone, while the relative amount of axial tubule (AT) components trended to increase slightly (Fig. 3C), in line with AT proliferation in cardiomyocyte pathophysiology^4,24,35^. The histograms in Fig. 3D point out the decrease in TT versus increase in AT components in myocytes of the MI border zone. Notably, electron tomography and 3D membrane reconstructions revealed residual AT components with lots of small membrane protrusions and intracellular vesicles in direct proximity to tubular membranes in the MI border zone (Fig. 3E), indicative of highly dynamic local tubular membrane remodelling processes. In summary, TAT membrane networks are degraded in cardiomyocytes of the MI border zone at the cost of TT components, whereas residual tubular membrane components show dynamic membrane protrusions and are surrounded by numerous intracellular vesicles. As intracellular vesicles next to TAT membranes were recently shown to contain dysferlin, we propose that dysferlin may mediate membrane remodelling and repair events at residual tubular membranes in cardiomyocytes of the MI border zone^24^.

### STED nanoscopy resolves large dysferlin clusters tightly decorating residual TAT membranes in cardiomyocytes of the MI border zone

**Fig. 4:**
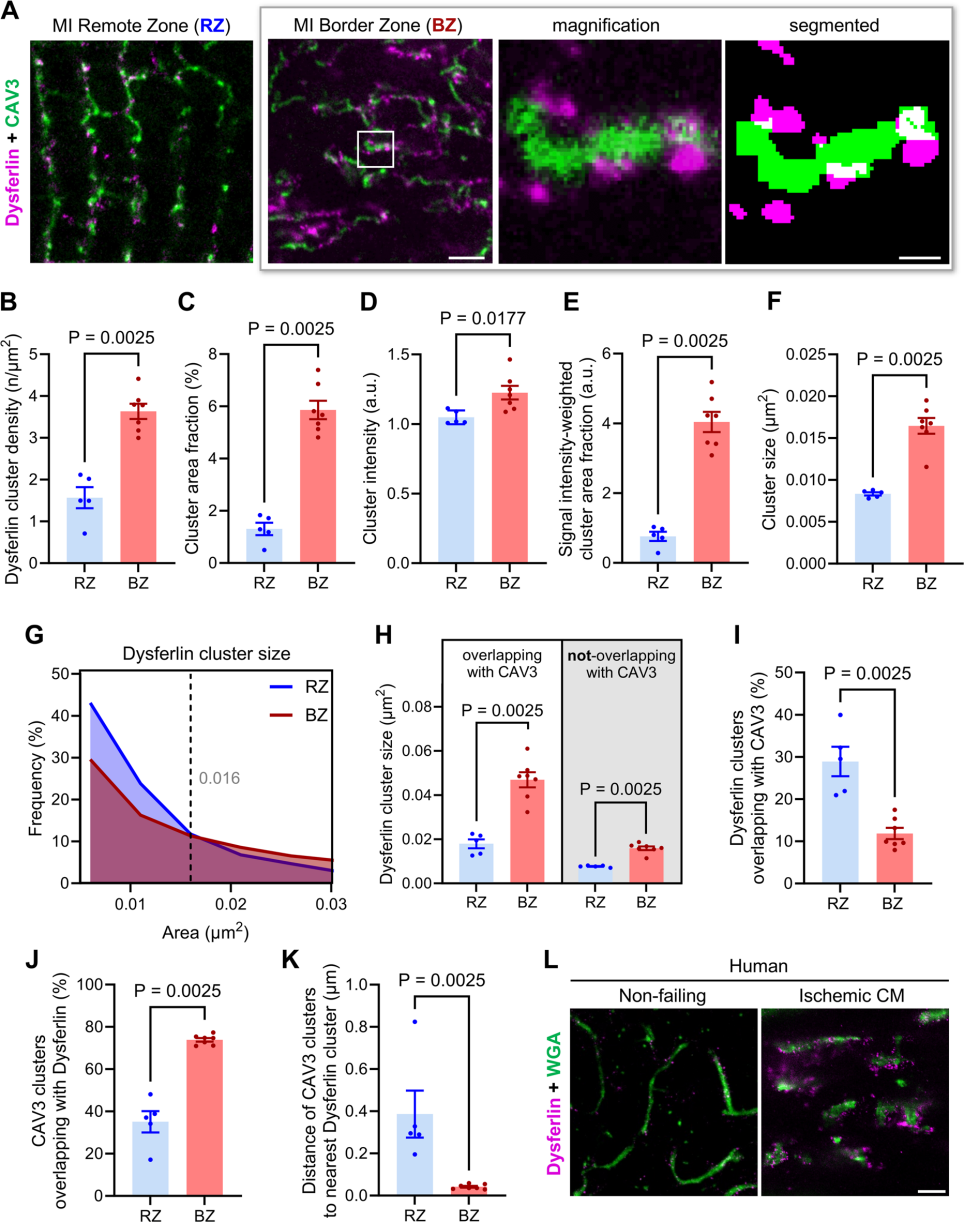
Enlarged dysferlin clusters tightly decorate residual TAT network structures in myocytes of the MI border zone. (**A**) Stimulated emission depletion (STED) imaging of dysferlin and the membrane and TAT network marker caveolin-3 (CAV3) in WT myocytes of the remote (RZ) and border zone (BZ) 1-week post-MI. In the border zone (BZ), dysferlin accumulations are identified in nanometric proximity to residual TAT network structures. *Right:* Single-channel signal segmentation was used for detailed dysferlin cluster analyses in (**B**–**L**). Scale bar 1 µm (left) and 10 nm (magnifications). (**B** through **E**), Intracellular dysferlin clusters in myocytes of the MI border zone show an increased cluster density (**B**), area fraction (**C**), and signal intensity (**D**) as compared to the remote zone, resulting in a significantly increased signal intensity-weighted cluster area fraction (**E**). (**F** through **H**), Dysferlin clusters in myocytes of the MI border zone are enlarged in size as confirmed by the dot plot in (**F**) and the frequency histogram in (**G**) (intersection point of frequency curves 0.016 µm^2^). Likewise, both types of dysferlin clusters, those directly overlapping and those *not*-overlapping with CAV3 signals are increased in size in the MI border zone as shown in (**H**). (**I** through **L**) While the relative number of dysferlin clusters overlapping with CAV3 signals is decreased in myocytes of the MI border zone (**I**), 74% vs 35% of CAV3 clusters overlap with dysferlin signals in the MI border compared to remote zone (**J**). In addition, the distance of CAV3 to dysferlin signals is markedly reduced in myocytes of the MI border zone as illustrated in the dot plot in (**K**). *n* = 43/66 LV myocytes of 5 RZ vs 7 BZ from individual mouse hearts in (**B**–**G**); and *n* = 43/47 LV myocytes of 5 RZ vs 7 BZ from individual mouse hearts in (**H**–**K**). Mann–Whitney *U* test. (**L**) STED imaging of dysferlin and the continuous membrane marker wheat germ agglutinin (WGA) at the TAT network of LV myocytes in biopsy sections from a human non-failing donor heart and a patient presented with ischemic cardiomyopathy (CM), representative of LV biopsies from 3 ischemic CM patients. Scale bar 1 µm.

To overcome the diffraction limitations of confocal microscopy, we applied STED imaging to resolve dysferlin signals near TAT structures 1-week post-MI at the nanoscale (Fig. 4, Figure S10A; see Figure S2 for dysferlin-KO used as immunofluorescence specificity control). As expected, STED imaging of dysferlin and caveolin-3 co-immunostained LV myocytes in tissue sections of the MI remote zone confirmed sporadic small dysferlin clusters in nanometric proximity to regularly organized caveolin-3-positive TT membrane signals along sarcomeric striations, with interconnecting AT components (Fig. 4A, left). In sharp contrast, residual TAT network components (mostly AT membranes) in cardiomyocytes of the MI border zone were tightly decorated by prominent dysferlin signals (Fig. 4A, lower right). Of note, the TAT network degradation in LV myocytes of the MI border zone was associated with disorganized and more fragmented SR Ca^2+^ release units as visualized by RyR2 immunostaining (Figure S10B), in line with decreased RyR2 and junctophilin-2 protein expression in the MI border zone (Figs. 1 and 2).

Dysferlin and caveolin-3 STED channels were then subjected to image segmentation protocols that allowed us to perform quantitative cluster analysis as previously described^24,35^. For this purpose, intracellular ROIs were manually selected, excluding dysferlin signals at the outer surface sarcolemma and the ICD membrane folds to set the focus on TAT membrane changes. (Details are outlined in the Supplemental Material.) Importantly, compared to cardiomyocytes of the remote zone, border zone cardiomyocytes exhibited twice the dysferlin cluster density (Fig. 4B), a fourfold increase in the cluster area fraction of dysferlin (Fig. 4C), and a small but significant increase in the mean cluster intensity (Fig. 4D), resulting in an overall fivefold increase of the dysferlin signal intensity-weighted cluster area fraction (Fig. 4E). Moreover, our analysis revealed a doubling of the mean dysferlin cluster size in MI border zone cardiomyocytes (Fig. 4F). The histogram in Fig. 4G illustrates the increase of larger dysferlin clusters at the expense of smaller signals (Fig. 4G; curve intersection at 0.016 µm^2^ cluster size).

Likewise, both types of dysferlin clusters, those directly overlapping and those not-overlapping with caveolin-3 signals were enlarged in the MI border zone, although dysferlin clusters overlapping with caveolin-3 were generally larger (Fig. 4H). The percentages of dysferlin clusters overlapping with caveolin-3 decreased in the MI border versus remote zone cardiomyocytes, presumably due to the TAT network degradation (Fig. 4I). Conversely, we found a strongly increased share of residual caveolin-3 signals overlapping with dysferlin (Fig. 4J). In general, a significantly smaller distance from caveolin-3 to dysferlin signals was observed in MI border zone cardiomyocytes (Fig. 4K). To test the relevance of our findings made in a mouse MI model, we studied LV biopsies from *n* = 3 human patients with ischemic cardiomyopathy compared to non-failing donor heart biopsies. Importantly, STED imaging of dysferlin and the continuous membrane marker wheat germ agglutinin in ischemic cardiomyopathy patient samples demonstrated accumulating and larger dysferlin clusters next to residual axial tubule membrane structures (Fig. 4L, Figure S10C). Taken together, an increased number of enlarged dysferlin clusters localizes in vicinity to residual tubular membrane structures in MI border zone cardiomyocytes. This supports the hypothesis that dysferlin functions as a Ca^2+^-sensitive membrane fusion and repair protein, involved in protecting the integrity and shaping the structure of TAT nanodomains post-MI.

### CoIP-based interactomic analysis identifies proteins of the ICD as novel dysferlin interaction partners

**Fig. 5:**
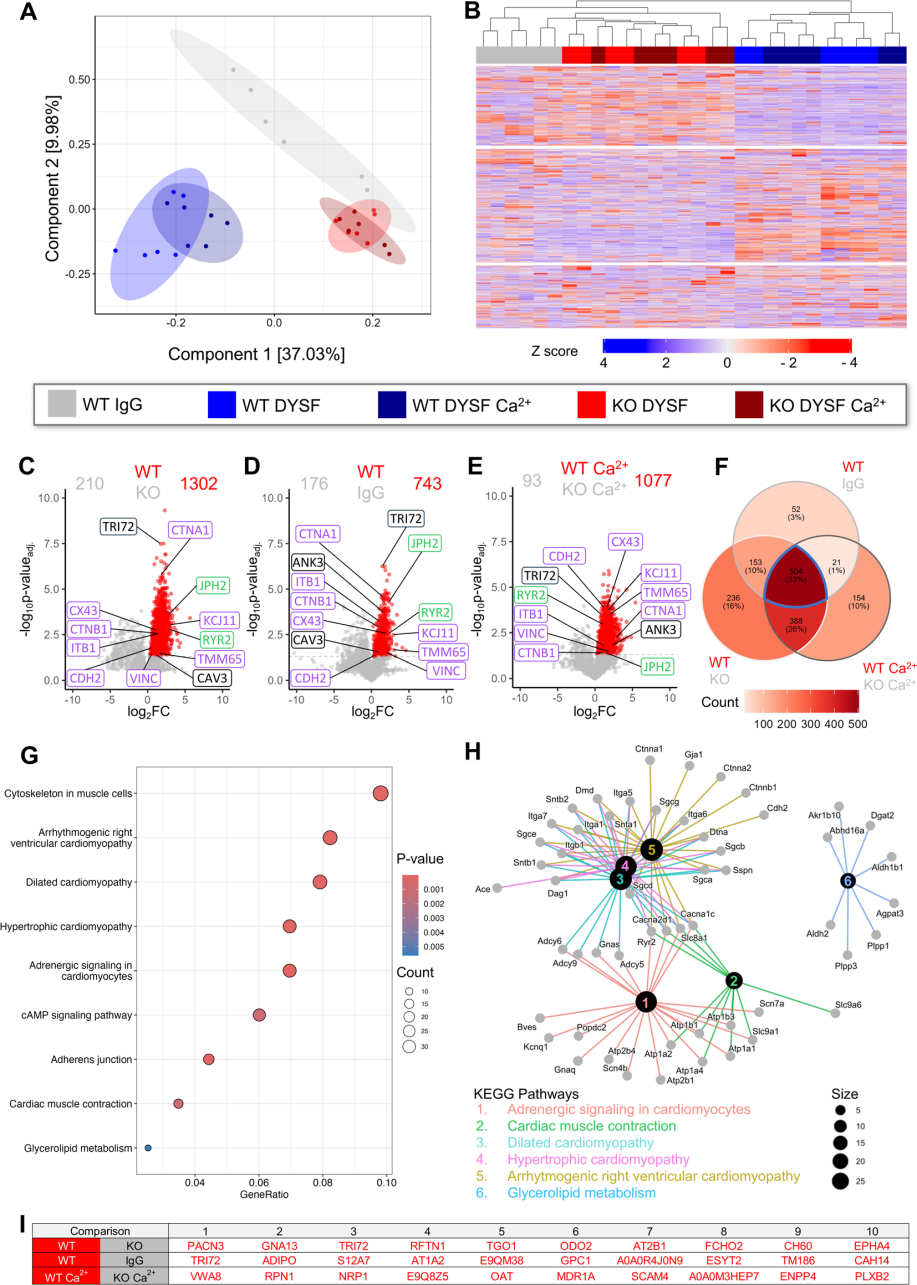
Dysferlin co-immunoprecipitation analysis in absence and presence of Ca^2+^ identifies novel interactions with ICD proteins. Eluates of dysferlin immunoprecipitation in absence and presence of 1 mmol/L [Ca^2+^] from left-ventricular mouse heart lysates were subjected to label-free DIA-MS for detailed dysferlin interactome studies. To control for unspecific protein binding, dysferlin-knockout (KO) samples and unspecific IgG controls were included. *n* = 3 individual mouse hearts analysed per group with two replicate injections per sample. (**A**) Principal component analysis (percentage of total variance listed in parentheses) confirms a clear separation of WT dysferlin co-immunoprecipitation (coIP, blue) vs control samples (red and grey). Ellipses indicate the 95% confidence intervals. (**B**) Heatmap with Euclidean clustering displaying 2,148 differentially enriched proteins as defined by blue-to-red colour table z score (ANOVA, FDR < 0.05). (**C** through **E**), Volcano plots with pairwise comparisons of coIPs from WT vs KO samples and IgG controls in absence and presence of Ca^2+^, respectively. The number of significantly precipitated proteins (*P* < 0.05) is indicated on top, and protein interaction candidates are indicated by red coloured dots. Selected protein interaction candidates are highlighted: ANK3, ankyrin-3; CAV3, caveolin-3; CHD2, cadherin-2; CTNA1, α-catenin; CTNB1, β-catenin; CX43, connexin-43; ITB1, integrin beta-1; JPH2, junctophilin-2; KCJ11, ATP-sensitive inward rectifier potassium channel 11; RYR2, ryanodine receptor type 2; TMM65; transmembrane protein 65; TRI72, tripartite motif-containing protein 72; VINC, vinculin. (**F**) Venn diagram visualizing the number of overlapping precipitated proteins among the 3 comparisons from (**C**–**E**). (**G** through **H**) KEGG pathway enrichment analysis. (**G**) Table highlighting 9 out of 63 KEGG pathways enriched by the dysferlin interactome found in all comparisons (504 proteins), with the gene ratio indicated by the size of the circles and the *P* value represented by the red-to-blue coloured lookup table. (**H**) Term-gene graph of 6 enriched KEGG pathways shown in (**G**). Term circle size indicates the number of precipitated proteins linked to each KEGG pathway. (**I**) Table summarizing the top 10 precipitated proteins in pairwise comparisons. All *P* values were adjusted for multiple testing using the Benjamini–Hochberg correction.

Previous studies demonstrated a substantial expression of dysferlin at ICD cell–cell contact sites of cardiomyocytes^24,36^, and suggested an interaction of dysferlin with ICD components^37^. Of note, our proteomic analysis revealed significant changes in the ICD gap junction organization in the WT versus dysferlin-KO MI border zone (Figure S5C). Hence, we aimed to clarify dysferlin’s localisation and interaction partners at ICD membrane folds before studying dysferlin’s pathophysiology at ICD membranes post-MI. Therefore, we employed coIP experiments with LV mouse heart tissue lysates based on an N-terminal dysferlin antibody in absence and presence of 1 mM [Ca^2+^], followed by DIA-MS analysis of the precipitates (Fig. 5). To control for unspecific protein binding, dysferlin-KO samples and unspecific IgG controls were included. Both the PCA plot and the heatmap with Euclidean clustering of all differentially precipitated proteins clearly segregated anti-dysferlin precipitates from dysferlin-KO and IgG controls, while anti-dysferlin precipitates with and without Ca^2+^ largely overlapped (Fig. 5A,B).

In Fig. 5C,D, volcano plots highlight 1,302 interaction candidates in the WT versus dysferlin-KO comparison (Fig. 5C), and 743 in the WT versus IgG comparison (Fig. 5D), both in absence of Ca^2+^. In presence of Ca^2+^, the WT versus dysferlin-KO comparison revealed 1,077 precipitated candidates (Fig. 5E). (All interaction candidates are specified in the *Supplemental Data Proteomics*.) The Venn diagram in Fig. 5F illustrates that 504 protein interaction partners were enriched in all 3 conditions (33%), while some specific candidates were only observed in one of the 3 comparisons (e.g., 154 proteins in the WT versus dysferlin-KO Ca^2+^ comparison).

Among dysferlin interaction candidates found in all 3 comparisons, we *i)* confirmed long-known interaction partners like caveolin-3 and tripartite motif-containing protein 72 (also known as MG53, coloured in *grey*)^32,38–40^, *ii)* detected proteins of the tubule-SR junction like RyR2 and junctophilin-2 that were recently identified as dysferlin interacting proteins (*green*)^24^, and *iii)* identified novel dysferlin interaction candidates that are well-known as integral components of the ICD nanodomain (*violet*, Fig. 5C–E). These include the gap junction protein connexin-43, the adherens junction protein N-cadherin, and further components of the adherens junction that mediate the coupling of N-cadherin to the contractile machinery like α-/β-catenin and vinculin (*violet*, Fig. 5C–E)^8^. As another interesting dysferlin interaction partner we detected the transmembrane protein 65 (TMM65, Fig. 5C–E), which was recently described as being critical for the structure and function of the ICD^41^. KEGG enrichment analysis identified 63 pathways based on the 504 interaction candidates found in all 3 comparisons, some of which were visualized in Fig. 5G. Corresponding term-gene graphs are shown in Fig. 5H. Additionally, KEGG pathways of singular comparisons are provided in Figure S11-15. Finally, Fig. 5I summarizes the top 10 precipitated proteins in pairwise comparisons. In conclusion, our coIP-based analysis revealed previously unknown dysferlin interacting proteins of both the adherens and gap junctions of the ICD membrane folds, which may provide the basis for local accumulations of dysferlin.

### Dysferlin localizes in close proximity to connexin-43 plaques and adherens junctions of the ICD membrane folds

**Fig. 6:**
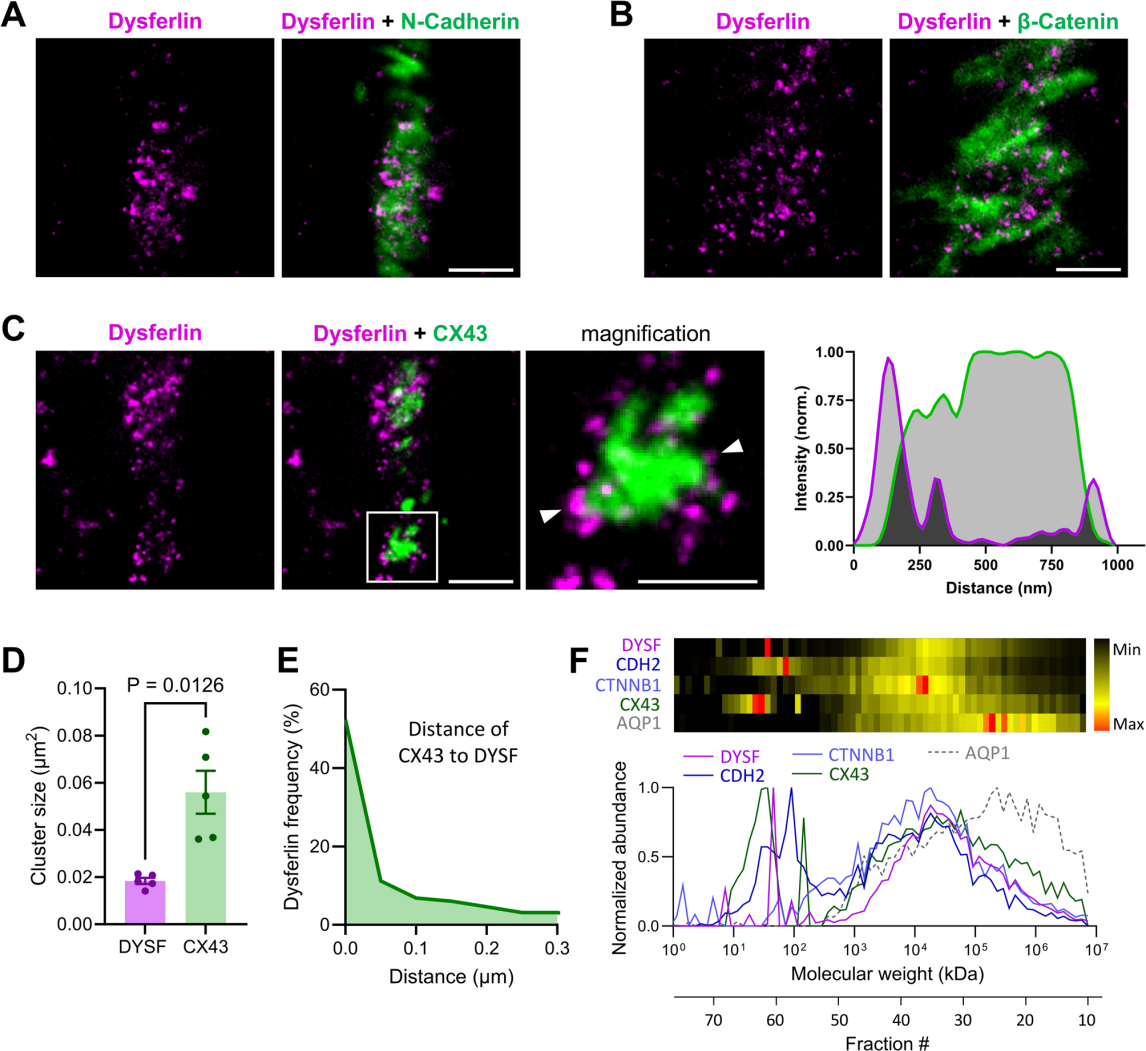
Dysferlin localizes in vicinity to and migrates in high-molecular weight complexes with ICD proteins. (**A** through **B**) STED imaging showing dysferlin in vicinity to the ICD proteins N-cadherin and β-catenin in cardiomyocytes of WT mouse left-ventricular tissue slices. Scale bars 1 µm. (**C**) STED imaging of dysferlin and the gap junction protein connexin-43 (CX43) identifies small dysferlin signals in nanometric proximity to large CX43 plaques at intercalated disc cell–cell contact sites. Scale bars 1 µm and 500 nm. Right, Signal intensity line profile illustrating the close spatial relationship of dysferlin and CX43 clusters in cardiomyocytes. (**D** through **E**) Detailed dysferlin and CX43 cluster analysis in WT LV myocytes. (**D**) Dysferlin clusters are on average 3 times smaller compared to CX43 clusters. Paired *t* test. (**E**) The relative frequency of dysferlin clusters grows exponentially with decreasing distance to the nearest CX43 cluster (bin centre-to-centre distances). *n* = 48 LV myocytes of 5 individual WT hearts in (**B**,**C**). (**F**) Complexome profiling of enriched membrane fractions from WT mouse myocardium reveals that dysferlin (DYSF), N-cadherin (CDH2), β-catenin (CTNNB1) and connexin-43 (CX43) co-migrate potentially as part of the same high molecular weight protein complex, which is distinct from the transmembrane protein aquaporin-1 (AQP1, negative control).

To clarify the spatial relationship of dysferlin to components of the ICD in detail, we applied STED imaging of co-immunolabelled WT LV cardiomyocytes. Indeed, STED co-immunofluorescence showed numerous small dysferlin clusters in direct proximity to the adherens junction proteins N-cadherin and β-catenin (Fig. 6A,B), both covering large segments of the ICD membrane folds. Additionally, several small dysferlin clusters were observed in vicinity to and surrounding large connexin-43 plaques (Fig. 6C, left), as highlighted by an intensity line profile (Fig. 6C, right). Quantitative cluster analysis after STED channel segmentation revealed that dysferlin signals at ICD membrane folds are three times smaller (Fig. 6D) but show a three times higher cluster density compared to connexin-43 plaques (Figure S16). Importantly, the histogram in Fig. 6E confirms that the relative frequency of dysferlin clusters grows rapidly with decreasing distance to the nearest connexin-43 plaque at ICD ROIs.

To determine whether dysferlin and ICD proteins form a native high molecular weight (MW) protein complex/supercomplex, we employed complexome profiling. Therefore, membrane pellets from WT mouse myocardium were homogenized under non-denaturating conditions and fractionated based on apparent MW via size exclusion chromatography followed by LC–MS/MS analysis^29^. Proteins which co-elute together in high-MW fractions may be inferred to be part of the same protein complex. Notably, dysferlin co-eluted together with N-cadherin, β-catenin and connexin-43, indicative of a high-MW protein complex (~ 10 MDa, Fig. 6F), which is distinct from other unrelated membrane protein complexes such as complexes involving aquaporin-1 (Fig. 6F) and transferrin receptor (not shown). In summary, we identified N-cadherin, β-catenin and connexin-43 as Ca^2+^-independent interaction partners of dysferlin, migrating in functional high MW protein complexes, and numerous small dysferlin clusters in local vicinity to adherens junctions and connexin-43 plaques at ICD membrane folds.

### Increased dysferlin expression stabilizes enlarged ICD cell–cell contact sites in the MI border zone

**Fig. 7:**
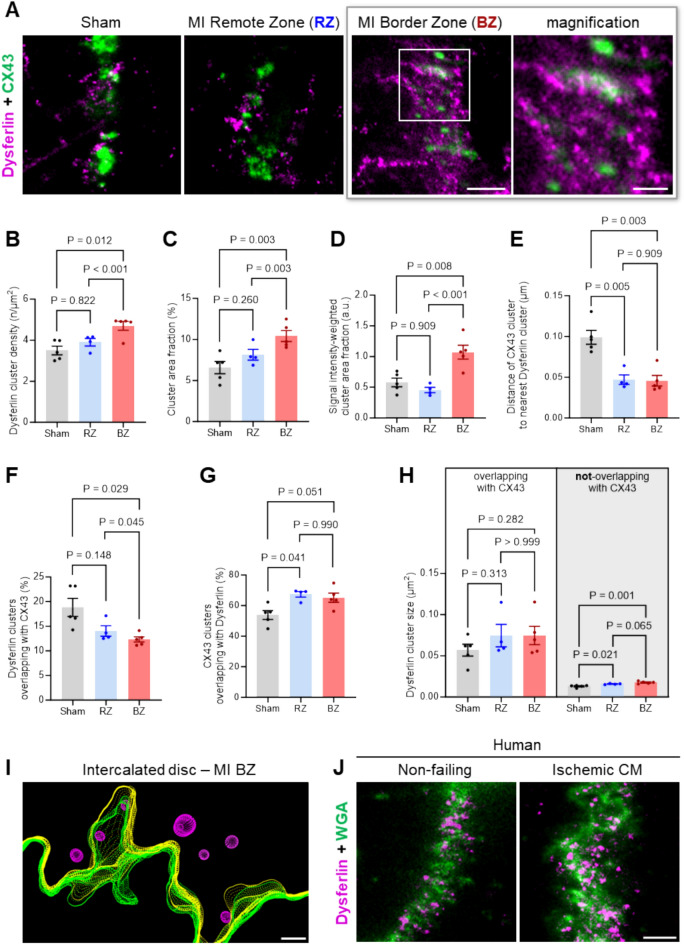
Dysferlin accumulates at enlarged intercalated disc cell–cell contact sites and connexin-43 plaques of myocytes in the MI border zone. (**A**) STED imaging of dysferlin and the gap junction protein connexin-43 (CX43) at intercalated disc (ICD) cell–cell contact sites of myocytes in the remote zone (RZ) and border zone (BZ) of WT mice 1-week post-MI vs sham-operated animals. Scale bars 1 µm and 500 nm. (**B** through **E**), Detailed cluster analysis reveals an increased dysferlin cluster density (**B**), area fraction (**C**), and signal intensity-weighted cluster area fraction (**D**) at the ICDs of myocytes in the MI border zone, while the distance of CX43 clusters to the nearest dysferlin cluster is significantly decreased in both the remote and border zone 1-week post-MI (**E**). (**F** through **G**), While the relative number of dysferlin clusters overlapping with CX43 is decreased, more CX43 clusters directly overlap with dysferlin at the ICDs of myocytes in the MI RZ and BZ. (**H**) Size of dysferlin clusters overlapping and not-overlapping with CX43. *n* = 48/43/47 LV myocytes of 5 sham controls, 4 RZ vs 5 BZ from individual mouse hearts in (**B**–**H**). Linear Mixed Model. (**I**) Electron tomography 3D reconstruction confirms numerous intracellular vesicles (magenta) in close proximity to the ICD membrane folds (yellow, upper cell; green, lower cell) in the MI border zone. Scale bar 200 nm. (**J**) STED imaging of dysferlin and the continuous membrane marker wheat germ agglutinin (WGA) at the ICD membrane folds of LV myocytes in biopsy sections from a human non-failing donor heart and a patient presented with ischemic cardiomyopathy (CM), representative of LV biopsies from 3 ischemic CM patients. Of note, ICD membrane folds are disorganized and enlarged in ischemic CM as compared to the non-failing control, and show more and larger dysferlin clusters. Scale bar 200 nm.

Due to their adjacency to the fibrotic infarct zone, cardiomyocytes of the MI border zone face a high biomechanical stress that may be translated to the ICD membrane folds, potentially requiring the membrane repair protein dysferlin for nanodomain stabilisation and repair. Supporting this hypothesis, significant abundance changes were observed for ICD proteins in our proteomic analysis post-MI, comparing the WT versus dysferlin-KO MI border zones (Fig. 2, Figure S5). Thus, we sought to analyse dysferlin’s expression and localization at ICD cell–cell contact sites in the border zone 1-week post-MI. Interestingly, STED co-immunofluorescence imaging of dysferlin and the membrane marker caveolin-3 recognized an enlarged ICD area in relation to the cardiomyocyte width and a 126% increased dysferlin signal intensity at ICD membrane folds in the MI border zone as compared to LV tissue slices from sham-treated animals (Figure S17A-C). STED imaging of dysferlin and connexin-43 confirmed the increased dysferlin signals at ICD membrane folds in the MI border zone versus remote zone and sham-treated myocardium (Fig. 7A, Figure S18A).

Quantitative analyses of STED channel segmentations uncovered a significant increase of the dysferlin cluster density (Fig. 7B), cluster area fraction (Fig. 7C), and signal intensity-weighted cluster area fraction (Fig. 7D) at ICD membrane folds in the MI border zone compared to the remote zone and sham myocardium. Concomitantly, we observed a smaller distance of connexin-43 plaques to the nearest dysferlin cluster in both MI border and remote zones (Fig. 7E), pointing toward a targeted co-localization of both proteins at ICDs in the post-MI setting. While the percentages of dysferlin clusters overlapping with connexin-43 plaques at ICDs declined based on the massive increase in dysferlin cluster density in the MI border zone (Fig. 7F), we found more connexin-43 plaques directly overlapping with dysferlin signals in both the MI remote and border zones (Fig. 7G). Dysferlin clusters overlapping with connexin-43 were generally larger than non-overlapping clusters independent of MI surgery (Fig. 7H).

Strikingly, electron tomography identified numerous intracellular vesicles in proximity to the ICD membrane folds in the MI border zone of mouse myocardium (Fig. 7I; Figure S18B), which may correspond to the increased dysferlin cluster density, given that dysferlin was previously localized in a compartment beneath the sarcolemma of cardiomyocytes^24^. To further investigate the significance of dysferlin clustering at ICD membranes in ischemic heart disease, we studied LV biopsies from *n* = 4 human patients with ischemic cardiomyopathy compared to non-failing donor heart biopsies. Confocal imaging confirmed an increased dysferlin signal intensity at ICD membranes in patients presented with ischemic cardiomyopathy (Figure S17D). STED imaging of dysferlin and the wheat germ agglutinin in ischemic cardiomyopathy patient samples showed enlarged ICD membrane folds with more and larger dysferlin clusters (Fig. 7J), confirming the relevance of our findings made in a mouse MI model for the human disease context. Taken together, enlarged ICD membrane folds in the MI border zone demonstrate a high local density of dysferlin clusters in proximity to and interacting with functionally relevant connexin-43 plaques, suggesting that dysferlin may stabilize the integrity of ICD cell–cell contact sites and potentially prevents progressive loss of function post-MI.

## Discussion

By applying pressure cycling technology tissue lysis and label-free DIA-MS for the analysis of small myocardial samples, we quantitatively analysed 5,700 proteins and revealed the proteomic nature of LV zones in the mouse heart post-MI. The DIA-MS dataset clearly segregated the differential proteotypes of the infarct, border and remote zones, thereby significantly extending the current knowledge of spatial remodelling mechanisms after MI (Fig. 2)^42^. Importantly, the absence of the membrane fusion and repair protein dysferlin significantly influenced the proteomic remodelling 1-week post-MI, as substantiated by 1,286 and 725 differentially abundant proteins in the WT versus dysferlin-KO comparisons of the infarct and border zone, respectively. Dysferlin itself showed an increased protein abundance in the border and remote zones, as confirmed by immunoblotting and immunohistology.

Subcellularly, confocal microscopy indicated a severe degradation of the TAT network in cardiomyocytes of the MI border zone at the cost of TT components, in line with loss of TAT membranes in the failing heart^6,43,44^. Capitalizing on the greater resolution of STED nanoscopy, we resolved unexpectedly large dysferlin signals in direct vicinity to residual TAT membranes of border zone cardiomyocytes, presumably supporting to seal, stabilize or shape TAT structures. Notably, almost every TAT component was directly decorated by intense dysferlin signals (Fig. 4). Our results were confirmed in LV biopsy samples from patients with ischemic cardiomyopathy. Electron tomography confirmed highly dynamic membrane protrusions of residual TAT components surrounded by numerous membrane vesicles, suggesting local membrane remodelling events.

At enlarged ICD membrane folds in border zone cardiomyocytes post-MI, the presence of local dysferlin expression doubled, which was confirmed in cardiomyocytes of small patient biopsies of ischemic cardiomyopathy. This might serve to maintain the integrity of the cell–cell contact sites. Interestingly, coIP-MS interactome analysis and complexome profiling revealed that the gap junction protein connexin-43 and the adherens junction proteins N-cadherin and β-catenin interact with dysferlin in functional high molecular weight complexes (Fig. 5). This likely explains the accumulation of dysferlin clusters beneath ICD membranes and provides a local basis for ICD sarcolemmal remodelling and repair events post-MI.

Finally, dysferlin deficiency was associated with significantly larger infarct size and reduced LV systolic function post-MI (Fig. 1). This may be attributed to the loss of critically stressed cardiomyocytes in the MI border zone of dysferlin-KO mice vis-a-vis a limited capacity of sarcolemmal nanodomain plasticity and repair.

In their study on the role of dysferlin in cardiac tissue, Han et al. were the first to investigate the effect of LAD ligation in WT versus dysferlin-KO animals^45^. In contrast to our work, the group did not observe significant differences between genotypes with respect to infarct size, ejection fraction, or the extent of left ventricular remodelling 1 day or 2 weeks post-surgery. However, only 6 WT versus 12 KO animals were included in their study, while our investigator-blinded echocardiographic measurements comprised 31 versus 21 animals (Fig. 1). Additionally, previous ex vivo experiments suggested an impaired recovery of dysferlin-KO hearts from acute ischemia/reperfusion (I/R) injury^21,22^, which was confirmed in vivo based on Masson’s trichrome staining by Evans et al.^23^ In line with those pre-existing I/R data, permanent LAD ligation in our dysferlin-KO animals led to larger infarct size, increased LV dilation and reduced LV contractility (Fig. 1). Although the I/R model may better reflect today’s acute care medicine with immediate reperfusion strategies^46^, we had to induce MI by permanent LAD ligation in this study to clearly demarcate the MI border zone from the infarct zone prior to region-specific proteomic DIA-MS analysis. Principally, dysferlin might have an even bigger effect in I/R compared to permanent LAD ligation, since I/R creates a larger area of critically stressed myocardium. Of note, our study was not powered to detect significant changes in WT versus dysferlin-KO mortality, as mortality is per se high post-MI and varies extremely between female and male animals^47^.

As visualized by immunohistology and based on increased dysferlin expression (Fig. 1D), the MI border zone is locally confined to a couple of cardiomyocyte rows lateral to the infarct zone. While the confined MI border zone complicates the isolation of cardiomyocytes for live-cell Ca^2+^ imaging, we would like to note that Hofhuis et al. previously showed a decreased L-type Ca^2+^ channel current versus increased spontaneous SR Ca^2+^ release events in isolated dysferlin-KO ventricular myocytes^48^. Nevertheless, the massive degradation of the TAT membrane network and the orphaning of RyR2 clusters in cardiomyocytes of the MI border zone will further impair the intracellular Ca^2+^ signalling (Fig. 3, Figure S10B), which is consistent with the enriched KEGG pathway ‘Ca^2+^-signalling’ in the WT versus KO MI border zone comparison (Figure S5C). Furthermore, data from skeletal muscle cells suggest that dysferlin may protect against pathological Ca^2+^ sparks and waves by buffering Ca^2+^ at residual tubule-SR junctions in cardiomyocytes of the MI border zone^49^, beyond its effects on membrane plasticity and repair. Future studies are required to investigate the effect of dysferlin deficiency on intracellular SR Ca^2+^ release, leak and buffering in cardiomyocytes of the MI border zone to identify potential therapeutic implications.

In line with dysferlin’s upregulation in LV pressure-overload induced by transverse aortic constriction^24^, non-ischemic myocardium of the MI remote zone presented a similar increase of dysferlin protein expression (Figs. 1 and 2). Whereas WT mice showed an increased anterior and posterior LV wall thickness in volume-overload post-MI as an indicator of compensatory LV hypertrophy, dysferlin-KO animals developed less LV hypertrophy compared to WT (Fig. 1E). Supposedly, dysferlin-mediated tubular membrane proliferation may be a prerequisite for pressure- and volume-overload induced cardiomyocyte hypertrophy and can be relevant for the outcome post-MI beyond its effect on the MI border zone. Together, these data underpin the relevance of dysferlin for the sarcolemmal plasticity of differentiated cardiomyocytes.

Among potential study limitations, we would like to point out that our co-immunofluorescence STED imaging protocol of dysferlin and caveolin-3 can only partly disclose the localization of dysferlin next to or directly on tubular/ICD membranes, particularly with the lack of a continuous membrane marker for mouse cardiomyocyte immunolabelling^50^. Furthermore, future studies are required to elucidate the precise dysferlin-positive vesicular compartment and the molecular mechanisms of dysferlin-mediated fusion and repair events of TAT and ICD membranes post-MI. We expect the presented interactomic data to guide such studies.

To maximize the number of proteins measured by DIA-MS in the MI border zone, we decided to excise the myocardial tissue post-MI under a binocular microscope using iris scissors. Here, laser microdissection represents a promising technique to analyse proteomic profiles of even smaller, better-defined myocardial regions or even single cell shapes in the future^51,52^.

In conclusion, our current findings suggest dysferlin as a key player to stabilize, repair and shape sarcolemmal nanodomains in cardiomyocytes of the MI border zone, namely the TAT endomembrane network and the ICD membrane folds, preventing from the loss of postmitotic cardiomyocytes and a further reduction in LV contractility post-MI. Future studies need to identify strategies for dysferlin activation or upregulation in patients who bear a high MI risk or suffer acutely from MI, in order to translate our findings into patient-tailored medical interventions.

## Supplementary Information

Supplementary Information 1.

Supplementary Information 2.

Supplementary Information 3.

## Data Availability

Proteomic data were deposited to the ProteomeXchange Consortium via the PRIDE partner repository^25^ with the dataset identifiers PXD060267 and PXD060259. All other data underlying this article will be shared upon reasonable request to the corresponding authors.

## Abbreviations

AT: Axial tubule
CoIP: Co-immunoprecipitation
DIA-MS: Data-independent acquisition mass-spectrometry
DYSF: Dysferlin
GO: Gene ontology
HIF-1: Hypoxia-inducible factor 1
ICD: Intercalated disc
I/R: Ischemia/reperfusion
KEGG: Kyoto encyclopedia of genes and genomes
KO: Knockout
LAD: Left anterior artery
LV: Left-ventricular
MG53: Mitsugumin-53
MI: Myocardial infarction
NF: Nonfailing
RyR2: Ryanodine receptor type 2
SR: Sarcoplasmic reticulum
STED: Stimulated emission depletion
TAT: Transverse-axial tubule
TT: Transverse tubule
VM: Ventricular myocyte
WT: Wild-type
