# Supplementary material for: Dysferlin stabilizes membrane nanodomains of cardiomyocytes after myocardial infarction
Source: Sci Rep. 2026 Mar 26;16:10488. doi: 10.1038/s41598-026-42800-9 (PMC13031966; doi:10.1038/s41598-026-42800-9)
Supplement: Supplementary file 1 — Supplementary Information 1. [file 41598_2026_42800_MOESM1_ESM.docx]

# Supplemental Material

**Dysferlin stabilizes membrane nanodomains of cardiomyocytes after myocardial infarction**

**Original Article**

**Authors:** Justus B. Wegener^1,2,3^†, Yannik Zühlke^1,2,3^†, Carolin Fleischhacker^1,2,4^, Justus Marks^1,2,3^, Brian Foo^1,2,3§^, Niklas Bader^1,2,4^, Gabriel C. Riedemann^1,2,3,4^, Jasper Wedemeyer^1,2,3^, Kim-Chi Vu^1,2^, Ana M. Vergel Leon^1,2,3,4^, Nora Josefine Paulke^1,2^, Tobias Kohl^1,2,4^, Henning Urlaub^3,7,9^, Constanze Schmidt^1,4^, Gerd Hasenfuß^1,3,4^, Tobias Moser^3,5,6^, Eva A. Rog-Zielinska^8^, Christof Lenz^3,4,7,9^, Stephan E. Lehnart^1,2,3,4^*, Sören Brandenburg^1,2,3,4^*

**Affiliations:**

^1^Department of Cardiology and Pneumology, University Medical Centre Göttingen; Göttingen, Germany.

^2^Cellular Biophysics & Translational Cardiology Section, Heart Research Centre, University Medical Centre Göttingen; Göttingen, Germany.

^3^Cluster of Excellence "Multiscale Bioimaging: from Molecular Machines to Networks of Excitable Cells" (MBExC), University of Göttingen; Göttingen, Germany.

^4^DZHK (German Centre for Cardiovascular Research), Partner Site Göttingen; Göttingen, Germany.

^5^Institute for Auditory Neuroscience and InnerEarLab, University Medical Centre Göttingen; Göttingen, Germany

^6^Auditory Neuroscience and Synaptic Nanophysiology Group, Max Planck Institute for Multidisciplinary Science, Göttingen, Germany

^7^Department of Clinical Chemistry, University Medical Centre Göttingen; Göttingen, Germany.

^8^Institute for Experimental Cardiovascular Medicine, University Heart Centre and Faculty of Medicine, University of Freiburg; Freiburg, Germany.

^9^Bioanalytical Mass Spectrometry Group, Max Planck Institute for Multidisciplinary Sciences; Göttingen, Germany.

* Corresponding authors

Robert-Koch-Straße 42a

37075 Göttingen, Germany

Tel. +49 551 39 62284

Fax +49 551 39 13063631

Emails: [soeren.brandenburg@med.uni-goettingen.de](mailto:soeren.brandenburg@med.uni-goettingen.de) and [slehnart@med.uni-goettingen.de](mailto:slehnart@med.uni-goettingen.de)

† JBW and YZ contributed equally to this work and are listed alphabetically.

# Supplementary Methods

Animal experiments and dysferlin-knockout mouse model

Myocardial infarction model

Echocardiography

Protein analysis by immunoblotting

Region-specific tissue proteomics using label-free DIA-MS after myocardial infarction

Cardiac dysferlin interactome

Confocal microscopy and STED nanoscopy

Quantitative immunohistology

Transverse-axial tubule network analysis

Intracellular dysferlin and caveolin-3 cluster analyses in LV myocytes post-MI

Dysferlin and connexin-43 cluster analyses at the intercalated disc cell-cell contact sites

Electron tomography

# Animal experiments and dysferlin-knockout mouse model

All experiments involving mice adhered to the guidelines outlined for the care and use of laboratory animals as per Directive 2010/63/EU of the European Parliament and NIH guidelines. Animal procedures followed institutional rules as reviewed by the local animal care and use committee, and were approved by the veterinarian state authority (LAVES, Oldenburg, Germany; animal protocol 33.9-42502-04-21/3785).

Unless stated otherwise, adult sex-mixed mice aged 8-16 weeks in the C57BL/6J background were used for experiments. This study used the dysferlin-knockout (KO) model as described previously.^1^ Breedings of heterozygous KO animals were realized to generate homozygous KO mice and littermate WT controls for all experiments. Ear punch genotyping via PCR was performed with the primer pairs 5’-GCCAGACAAGCAAGGTTAGTGTGG-3‘ and 5‘‑GCGGGCTCTCAGGCACAGTATCTGC-3‘ for the wild-type (WT) allele (resulting in a 3400 bp PCR product), and 5’-GCCAGACAAGCAAGGTTAGTGTGG-3’ and 5’‑GCTGACTCTAGAGCTTGCGGAACC-3’ for the KO allele (resulting in a 3000 bp PCR product).

# Myocardial infarction model

For myocardial infarction (MI) of the anterior wall, cohorts of mice aged 8 to 15 weeks underwent surgical ligation of the left anterior descending (LAD) artery. Successful occlusion was confirmed by prompt livid color change observed in the myocardium distal to LAD ligation. Access to the heart was obtained through left thoracotomy. The thoracic cavity was opened through a small incision in the 4^th^ intercostal space, and the pericardium was surgically removed to prevent fibrotic processes.

For analgesia, carprofen was injected subcutaneously 30 min before surgery. Anaesthesia was administered to the mice through intraperitoneal injections of medetomidine (0.5 mg/kg bodyweight), midazolam (5 mg/kg bodyweight), and fentanyl (0.05 mg/kg bodyweight) prior to intubation of the trachea. Throughout the ~ 15 min surgical procedure, mice were positioned on a 37°C warming plate. At the end of surgery, atipamezole (2.5 mg/kg bodyweight) and flumazenil (0.5 mg/kg bodyweight) were administered subcutaneously to counteract residual sedation, and buprenorphine (0.05-0.1 mg/kg bodyweight) to alleviate postoperative pain. Monitoring during the procedure included the tracking of body temperature, respiration rate, and surface ECG. Following surgery, mice were housed in a cage positioned on a warming plate for 24 h. The day after surgery, carprofen (5 mg/kg bodyweight) was administered routinely, and additional analgesia was provided if recommended by local veterinarians.

Sham-operated animals were treated in the same manner, without ligation of the LAD.

# Echocardiography

Transthoracic echocardiography of adult mice was performed under mild anaesthesia (1.5% isoflurane in oxygen). B-mode and corresponding M-mode images were acquired in the parasternal long-axis view, encompassing the aortic valve to the endocardium of the cardiac apex, as well as in the parasternal short-axis view using a 30 MHz transducer (Vevo 2100, VisualSonics). Throughout echocardiography, body temperature, respiration rate, and surface ECG traces were continuously monitored, and a red-light lamp was employed to maintain body temperature. Investigators and data analysts were blinded to the treatment (MI vs sham surgery) and the genotype of the mice. The midventricular parasternal short axis view was used to determine the infarct size of WT vs KO mice 1 week post-MI, defined by the extent of the akinetic left ventricular wall.

# Protein analysis by immunoblotting

Mouse hearts were retrogradely perfused with 0.9% NaCl at 37°C for 2 min using a modified Langendorff setup, effectively removing blood from the myocardium. Left-ventricular tissues were dissected under a binocular microscope, promptly snap frozen in liquid nitrogen, and stored at ‑80°C. Immunoblots of left-ventricular tissues from MI mice excluded the infarct zone unless stated otherwise. Cardiac tissues were homogenized in ice-cold homogenization buffer using a 1‑D Ultra Turrax (Miccra). Supplementary Table 2 provides detailed information about the buffer solutions used. Homogenates were solubilized for 20 min by rotation at 4°C and subsequently centrifuged at 8,000 g for 10 minutes at 4 °C to obtain the post-nuclear fraction. The protein concentration of the supernatant was determined using the Pierce BCA-protein assay (Thermo Fisher Scientific). For immunoblotting, 20 µg of protein per lane were separated by SDS-PAGE on 4-20% Tris-HCl gradient gels. Proteins were transferred onto PVDF membranes (0.45 µm pore size, Immobilon-FL, Merck Millipore) at a constant 100 V for 2 hours at 4°C in transfer buffer. Subsequently, membranes were blocked in 5% w/v non-fat milk in TBST (blocking buffer) for 1 hour at 20 °C.

Primary antibodies (refer to Supplementary Table 1 for detailed information) were diluted in blocking buffer and incubated overnight at 4°C. Following washing, blots were incubated with fluorescent anti-mouse and anti-rabbit secondary antibodies (donkey anti-rabbit P/N 926-68073 and P/N 926-32213; and donkey anti-mouse P/N 926-68072 and P/N 926-32212, IRDye LICOR) diluted 1:15,000 in 10% ROTI®Block (A151.4, Carl Roth) and TBST for a minimum of 2 h. Protein detection was performed using the Odyssey CLx imaging system (LI-COR), and band densities were analysed using Image Studio lite version 5.2. Proteins were normalized to GAPDH.

# Region-specific tissue proteomics using label-free DIA-MS after myocardial infarction

After heart extraction and retrograde myocardial perfusion as mentioned above, left ventricles of 5 WT vs 5 KO mice 1 week post-MI were carefully dissected into infarct zone, border zone and remote zone using a binocular microscope (Zeiss Stemi 305). The MI border zone was defined as small lamella directly lateral to the infarct zone. Samples from sham-operated animals were acquired in the same manner. The small myocardial samples were lysed in 2% SDS, 100 mM HEPES buffer in a Barocycler 2320EXT (Pressure Biosciences), tryptically digested using an SP3 protocol on amine-coated paramagnetic beads (Resyn Bioscience), and analysed using label-free data-independent acquisition mass spectrometry (DIA-MS) performed on a timsTOF Pro 2 (Bruker) mass spectrometer with two replicate injections per sample (400 ng equivalent loaded, 100 min gradient, 20x2 variable window diaPASEF method). Additional identifications were obtained using data-dependent acquisition (DDA) on the timsTOF Pro 2 (Bruker) (400 ng equivalent loaded, 100 min gradient, ddaPASEF method, 12 samples). Raw data were processed by Biognosys Spectronaut v16.0.220606.53000. The combined qualitative runs were searched against the UniProtKB mouse reference proteome v1.2021 with default parameters at a FDR of 1%. Across all samples, 91,938 precursors corresponding to 65,979 stripped peptide sequences were detected and quantified, and combined to 5,700 protein group abundance values, which were normalized by quartile normalization after global data imputation. Spectronaut data were further analysed using R v4.3.1 (R Core Team (2023) R: A Language and Environment for Statistical Computing. R Foundation for Statistical Computing, Vienna. <https://www.R-project.org/>). Principal Component Analysis was applied utilizing the R package stats v4.3.1. Proteins with significant changes between experimental groups as determined by ANOVA underwent z-score normalization and hierarchical clustering with Euclidean distance, employing the R package ComplexHeatmap v2.13.1.^2^ Group comparisons were performed using the Welch *t* test with Benjamini-Hochberg correction for multiple testing (FDR threshold 0.05). Results were presented in volcano plots with the R package tidyverse v2.0.0,^3^ and underwent gene ontology analysis using the R package clusterProfiler v4.8.3,^4^ excluding blood related proteins with the gene ontology annotations GO:0007596, GO:0072562 and GO:0016064. KEGG pathway enrichment analyses were performed using pathfindR v2.4.1.^5^

# Cardiac dysferlin interactome

Left-ventricular myocardium from six wild-type and three KO mice, after washing using a modified Langendorff setup to remove myocardial blood as described above, were homogenized using a 1-D Ultra Turrax (Miccra). Homogenates were centrifuged at 5,000 g and 4 °C for 10 min to eliminate cell debris. The supernatant was used for membrane preparation through ultracentrifugation at 100,000 g and 4°C for 1 h. The pellet was resolubilised in 1 mL lysis buffer in absence or presence of 1 mmol/L [Ca^2+^] (please refer to Supplementary Table 2 for detailed buffer compositions), and the protein concentration was determined using the Pierce BCA protein assay (Thermo Fisher). For coimmunoprecipitation (CoIP) of dysferlin protein interaction candidates, 500 µg of protein lysate were incubated with 4 µg of dysferlin antibody (ab124684, Abcam) or 4 µg of unspecific rabbit IgG (12-370, Millipore) as a negative control in a total volume of 1 mL lysis buffer overnight under rotation at 4 °C. After washing, 50 µL of a 50% slurry of magnetic beads (Dynabeads Protein G, Thermo Fisher Scientific) were added to the lysate-antibody mix and incubated for another 1.5 h at 4 °C under rotation. Subsequently, samples were placed on a magnet (DynaMag2 magnet, Thermo Fisher Scientific) to obtain the beads-antibody-antigen complexes. Samples underwent three 10 min washing steps with 1 mL lysis buffer under rotation to minimize unspecific binding. The precipitated beads-antibody-antigen complexes were ultimately eluted in fresh tubes by 70 µL of 2x SDS sample buffer. After vigorous mixing at 95°C for 10 minutes, the supernatant was collected for DIA-MS analysis. CoIP samples were purified by short-run SDS-PAGE and tryptically digested in-gel. Quantification of peptides by data-independent acquisition (DIA) used the Bruker timsTOF Pro 2 (250 ng equivalent loaded, 60 min gradient, 16x2 variable window diaPASEF method, 2 technical replicates per sample). Raw data were processed in Biognosys Spectronaut v16.0.220606.53000. Protein identification and spectral library generation from DIA used the Pulsar search engine against UniProtKB mouse reference proteome v1.2021 with default parameters, all at 1% FDR. DIA quantification used up to 6 fragments per peptide, and up to 10 peptides per protein. Dynamic retention time alignment and dynamic mass recalibration was performed at 1% FDR. The resulting data was imputed globally and normalized using quartile normalization. Spectronaut data were further analyzed as described in the section above (Label-free quantitative mass spectrometry of mouse heart tissue). Venn diagrams were created with the R package ggVennDiagramm v1.5.0 (<https://cran.r-project.org/web/packages/ggVennDiagram/index.html>; doi: 10.32614/CRAN.package.ggVenn Diagram)

# Confocal microscopy and STED nanoscopy

Sample preparation for immunohistology is outlined in detail in the main Methods section. Confocal and STED imaging of LV mouse and human heart tissue was applied using a Leica TCS SP8 system equipped with a HC PL APO C2S 100x/1.40 oil, HC PL APO CS2 40x/1.30 oil and HC PL FLUOTAR 10x/0.30 dry objective. STED workflow was optimized for the STAR 635P and STAR 580 fluorophores, employing the following microscope settings: pixel size 16.23 x 16.23 nm, pixel dwell time 400 ns, scanning speed 600 Hz, 16x line averaging, excitation with a white-light laser at 635 and 580 nm, STED depletion at 775 nm, and fluorescence detection in the ranges of 650-700 nm and 600-630 nm. Raw images were processed using Fiji (<https://imagej.net/Fiji>).

# Quantitative immunohistology

For quantitative dysferlin intensity analysis of immunolabeled mouse midventricular heart sections 1- and 4-weeks post-MI vs sham controls, we acquired confocal images of dysferlin and the cardiomyocyte membrane and transverse-axial tubule marker caveolin-3 with the HC PL FLUOTAR 10x/0.30 dry objective, where infarction, border and remote zone were optimally presented. ROIs were manually defined in Fiji (<https://imagej.net/Fiji>). The infarct zone was easily identified by absence of robust caveolin-3 signal. The border zone was specified as the first 3 lines of myocytes lateral to the infarct zone. The transition zone was defined as the 5 lines of myocytes next to the border zone, and the myocardium beyond the transition zone was specified as MI remote zone. For sham controls (i.e. same surgery but no LAD ligation), we defined ROIs including myocytes of the LV anterior wall. Spatial dysferlin intensities were normalized to sham controls.

# Transverse-axial tubule network analysis

Confocal images of caveolin-3 immunolabeled midventricular sections of mouse hearts 1-week post-MI vs sham controls were acquired with the HC PL APO C2S 100x/1.40 oil objective. Intracellular myocyte ROIs were manually defined excluding the lateral surface membrane and the nuclei, and the main cell axes were horizontally aligned for detailed transverse-axial tubule (TAT) network analysis. For network segmentation, caveolin-3 signals were background subtracted (rolling ball radius 5 pixels), local contrast enhanced (CLAHE; block size 49, histogram bins 256, max slope 3, mask none), smoothed (Gaussian blur, sigma 1.2 pixels) and merged (Statistical region merging, q=100 showaverages). Next, a manual threshold (60-255, 8-bit format) was used for signal binarisation. Binary images were then skeletonized and analysed in Fiji using the "Analyze Skeleton (2D/3D)" plugin to calculate TAT network density and branch length. Additionally, the orientation of caveolin-3 labelled TAT network components was assessed using the "Directionality" Fiji plugin. Given the horizontal orientation of the main cell axis, axial tubules (AT) correspond to 0°, while transverse tubules (TT) are defined by 90° in the TAT network composition histograms.

# Intracellular dysferlin and caveolin-3 cluster analyses in LV myocytes post-MI

For quantitative analysis of dysferlin and caveolin-3 cluster properties based on dual-colour STED immunofluorescence imaging, we adapted previously published protocols^7^ in Fiji (<https://imagej.net/Fiji>). Intracellular ROIs excluding the surface membrane and the nuclei were manually defined. The immunofluorescence signal patterns were segmented as described below:

1. Dysferlin channels (fluorophore STAR 635P) were background subtracted (rolling ball radius 30 pixels), local contrast enhanced (CLAHE; block size 30, histogram bins 256, max slope 3, mask none), and smoothed (Gaussian blur, sigma 1 pixel). Finally, auto local Bernsen thresholding (radius 20 pixels) was used for signal binarisation.
2. Caveolin-3 channels (fluorophore STAR 580) were background subtracted (rolling ball radius 30 pixels), local contrast enhanced (CLAHE; block size 49, histogram bins 256, max slope 3, mask none), smoothed (Gaussian blur, sigma 2 pixels) and merged (Statistical region merging, q=100 showaverages). Next, a manual thresholding (60,265) was used for signal binarisation.

Subsequently, segmented dysferlin images were used to analyse single clusters in order to determine cluster size, density (number of clusters per µm^2^) and the signal area fraction (total area of clusters per µm^2^). Only clusters > 0.0006 µm^2^ were included into further analyses. For overlap analysis of dysferlin and caveolin-3, we adapted previously published protocols^7^ and accepted partial to complete overlapping clusters in segmented images. Segmented images were also used to generate distance maps, determining the nearest neighbour distance from protein clusters in channel 2 to clusters in channel 1.

# Dysferlin and connexin-43 cluster analyses at the intercalated disc cell-cell contact sites

STED images of highly interdigitated intercalated disc (ICD) membrane folds were deconvoluted using Huygens 19.04.0p5. For quantitative analysis of dysferlin and connexin-43 cluster properties based on dual-colour STED immunofluorescence imaging, ROIs were manually defined encompassing the ICD cell-cell contact sites. The immunofluorescence signal patterns were segmented as follows:

1. Dysferlin channels (fluorophore STAR 635P) were background subtracted (rolling ball radius 30 pixels), local contrast enhanced (CLAHE; block size 30, histogram bins 256, max slope 3, mask none), and smoothed (Gaussian blur, sigma 1 pixel). Finally, auto local Bernsen thresholding (radius 20 pixels) was used for signal binarisation.
2. Connexin-43 channels (STAR 580) were background subtracted (rolling ball radius 45 pixels), local contrast enhanced (CLAHE; block size 45, histogram bins 256, max slope 3, mask none), smoothed (Gaussian blur, sigma 2 pixels) and merged (Statistical region merging, q=100 showaverages). Afterwards, auto local Bernsen thresholding (radius 35 pixels) was used for signal binarisation.

Subsequently, segmented dysferlin and connexin-43 images were used to analyse single clusters in order to determine cluster size, density (number of clusters per µm^2^) and the signal area fraction (total area of clusters per µm^2^). Only dysferlin clusters > 0.0006 µm^2^ and connexin-43 clusters > 0.001 µm^2^ were included into further analyses. Overlap and nearest neighbour distance analyses were performed as described above for dysferlin/caveolin-3.

# Electron tomography

Mouse hearts post-MI (*n*=5) vs sham surgery (*n*=3) were perfusion‐fixed with isoosmotic Karnovsky's fixative (2.4% sodium cacodylate, 0.75% paraformaldehyde, and 0.75% glutaraldehyde; 300 mOsm). Tissue fragments were excised from the left-ventricular MI border zone and washed with 100 mM sodium cacodylate, post‐fixed in 1% OsO_4_ for 1 h, dehydrated in graded acetone, and embedded in Epon‐Araldite resin. Semi‐thick sections (300 nm) were placed on formvar‐coated copper slot‐grids, post-stained with 2% aqueous uranyl acetate and Reynold's lead citrate. Sections were imaged using 300 kV Tecnai TF30 (FEI Company, now Thermo-Fisher Scientific, Eindhoven, The Netherlands). Tilt series were aligned, reconstructed, and combined using IMOD as described previously.^8^

# Supplemental Figures

Supplementary Figure 1. Immunoblot of left-ventricular mouse heart lysates after myocardial infarction.

Supplementary Figure 2. Dysferlin immunofluorescence signal specificity control.

Supplementary Figure 3. Baseline echocardiography prior to myocardial infarction surgery.

Supplementary Figure 4. Survival analysis of wild-type and dysferlin-knockout mice after myocardial infarction.

Supplementary Figure 5. Comparison of enriched KEGG pathways in wild-type vs. dysferlin-knockout MI zone comparisons.

Supplementary Figure 6. PathfindR analysis of KEGG pathways in the left-ventricular infarct zone of wild-type and dysferlin-knockout mice.

Supplementary Figure 7. PathfindR analysis of KEGG pathways in the left-ventricular border zone of wild-type and dysferlin-knockout mice.

Supplementary Figure 8. PathfindR analysis of KEGG pathways in the left-ventricular remote zone of wild-type and dysferlin-knockout mice.

Supplementary Figure 9. Representative confocal co-immunofluorescence images of dysferlin and caveolin-3 in left-ventricular tissue sections from wild-type mice post-MI.

Supplementary Figure 10. Dysferlin accumulations at residual TAT structures in cardiomyocytes of the MI border zone.

Supplementary Figure 11. PathfindR analysis of KEGG pathways of the anti-dysferlin co-immunoprecipitation from left-ventricular myocardial lysates compared with unspecific IgG control.

Supplementary Figure 12. PathfindR analysis of KEGG pathways of the anti-dysferlin co-immunoprecipitation from wildtype vs. dysferlin-knockout left-ventricular myocardial lysates.

Supplementary Figure 13. PathfindR analysis of KEGG pathways of the anti-dysferlin co-immunoprecipitation from wildtype vs. dysferlin-knockout left-ventricular myocardial lysates in presence of 1 mM [Ca^2+^].

Supplementary Figure 14. PathfindR term-gene graph of KEGG pathways of the anti-dysferlin co-immunoprecipitation from wildtype vs. dysferlin-knockout left-ventricular myocardial lysates.

Supplementary Figure 15. PathfindR analysis of KEGG pathways from proteins only enriched in presence of 1 mM [Ca^2+^] in the anti-dysferlin co-immunoprecipitation from wildtype vs. dysferlin-knockout left-ventricular myocardial lysates.

Supplementary Figure 16. Cluster density of dysferlin and connexin-43 at the intercalated disc membrane folds.

Supplementary Figure 17. STED co-immunofluorescence imaging of dysferlin and Caveolin-3 at the intercalated discs in cardiomyocytes of the MI border zone.

Supplementary Figure 18. STED co-immunofluorescence imaging of dysferlin and connexin-43 at the intercalated discs in cardiomyocytes of the MI remote and border zone.

Supplementary Table 1. Primary antibodies used in immunoblotting and immunofluorescence imaging as referenced in the figure legends or methods section.

Supplementary Table 2. Composition of buffer solutions as referenced in the methods section in mmol/L.

Supplementary Table 3. Left ventricular mouse echocardiography 1 week post-MI.


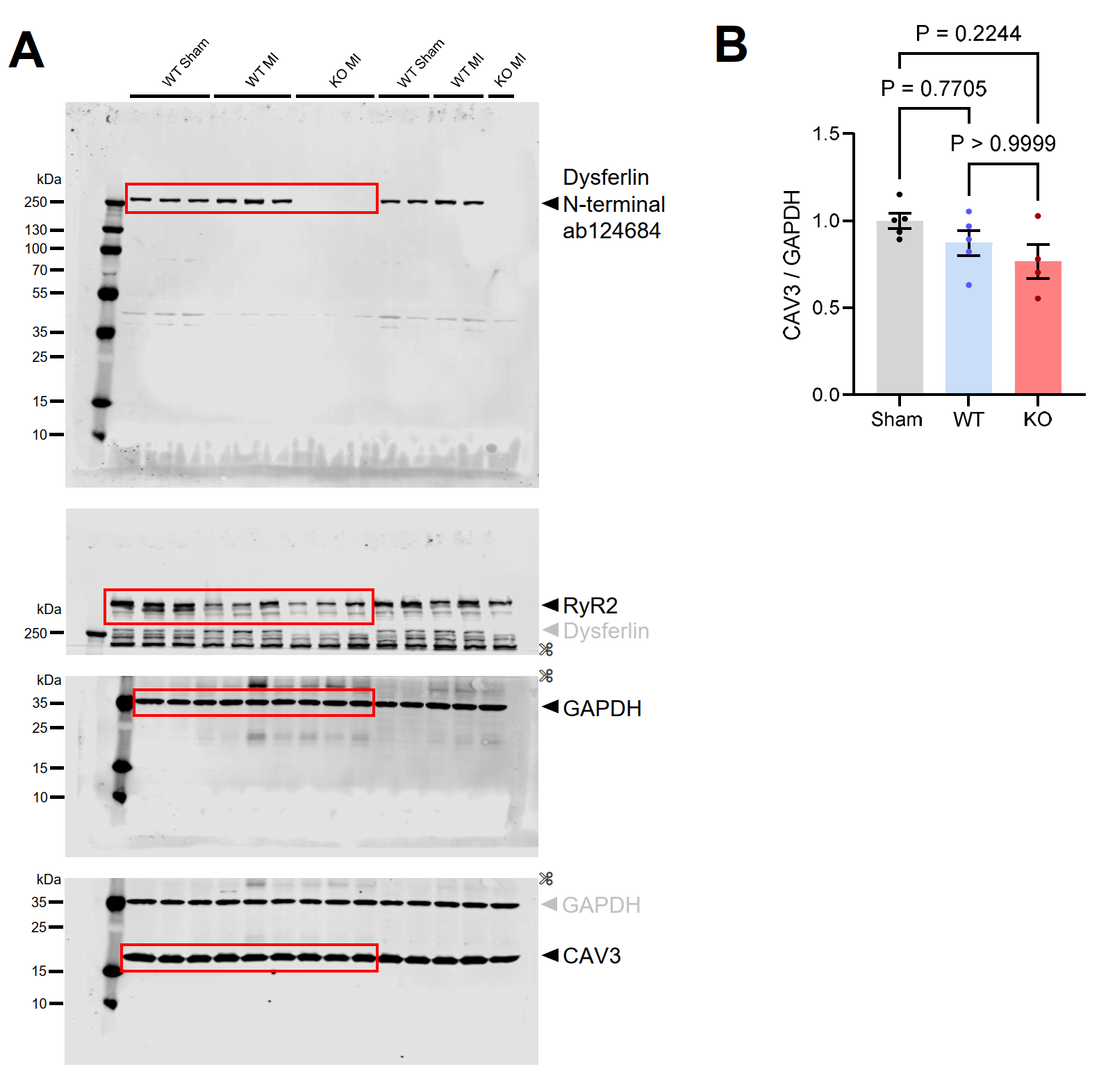


# Supplementary Figure 1. Immunoblot of left-ventricular mouse heart lysates after myocardial infarction.

**A**, Full immunoblots of LV mouse heart lysates 1 week after myocardial infarction (MI) or sham surgery. Infarct zones were excised from left ventricles prior to sample lysis. Red boxes indicate the cropped images shown in main Figure 1. After dysferlin detection, full-length membrane was cut (✂) to allow for incubation with multiple primary antibodies from the same species. WT, wild-type. KO, Dysferlin knockout. **B**, Analysis of the caveolin-3 (CAV3) immunoblot signals shown in **A**, normalized to GAPDH. *n*=5 WT sham, 5 WT MI vs 4 KO MI hearts. Kruskal-Wallis test.


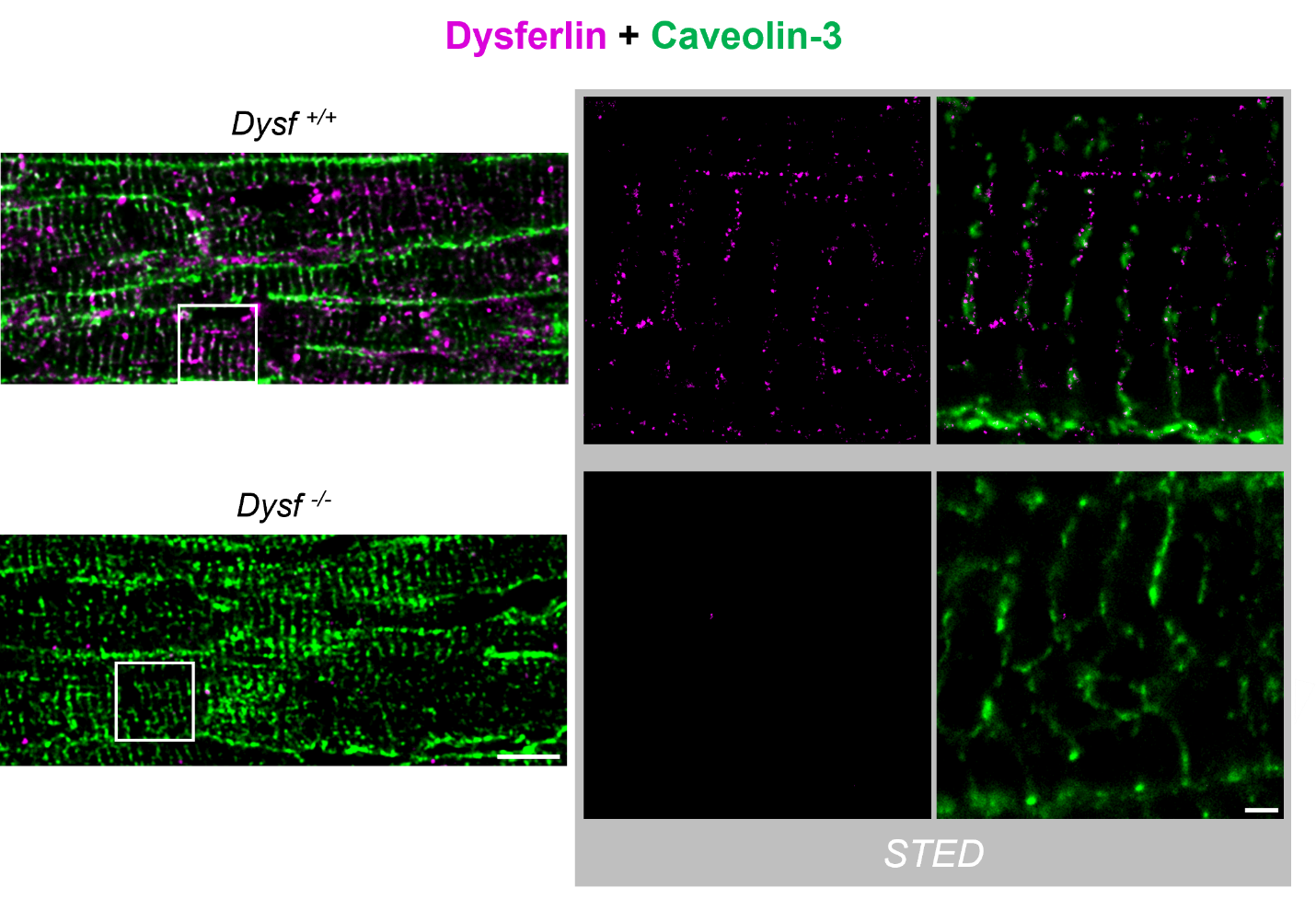


# Supplementary Figure 2. Dysferlin immunofluorescence signal specificity control.

Confocal (left) and stimulated emission depletion (STED, right) co-immunofluorescence images of dysferlin (magenta) and the membrane and transverse-axial tubule (TAT) marker caveolin-3 (green) in LV tissue sections from wild-type (*Dysf ^+/+^*) versus Dysferlin-knockout (*Dysf ^-/-^*) hearts. White boxes highlight regions magnified on the right. Scale bar 10 µm (cell overviews), and 1µm (magnifications).


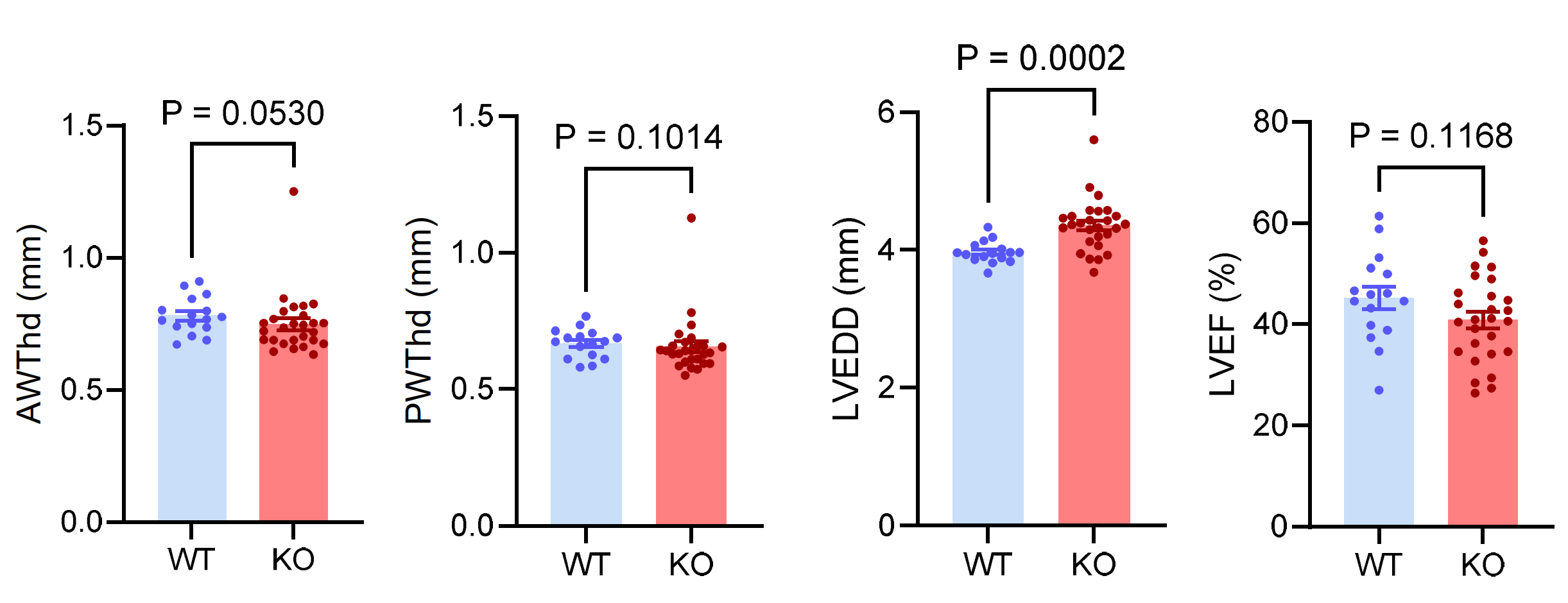


# Supplementary Figure 3. Baseline echocardiography prior to myocardial infarction surgery.

Transthoracic echocardiography comparing LV dimensions and systolic function of wild-type (WT) vs dysferlin-knockout (KO) mice prior to MI surgery at an age of 10 weeks. AWThd, anterior wall thickness in diastole; LVEDD, left-ventricular enddiastolic diameter; LVEF; left-ventricular ejection fraction; PWThd, posterior wall thickness in diastole. *n*=16 WT vs 27 KO mice. Unpaired Mann-Whitney *U* test (AWThd, PWThd, LVEDD) and unpaired Welch *t* test (LVEF).


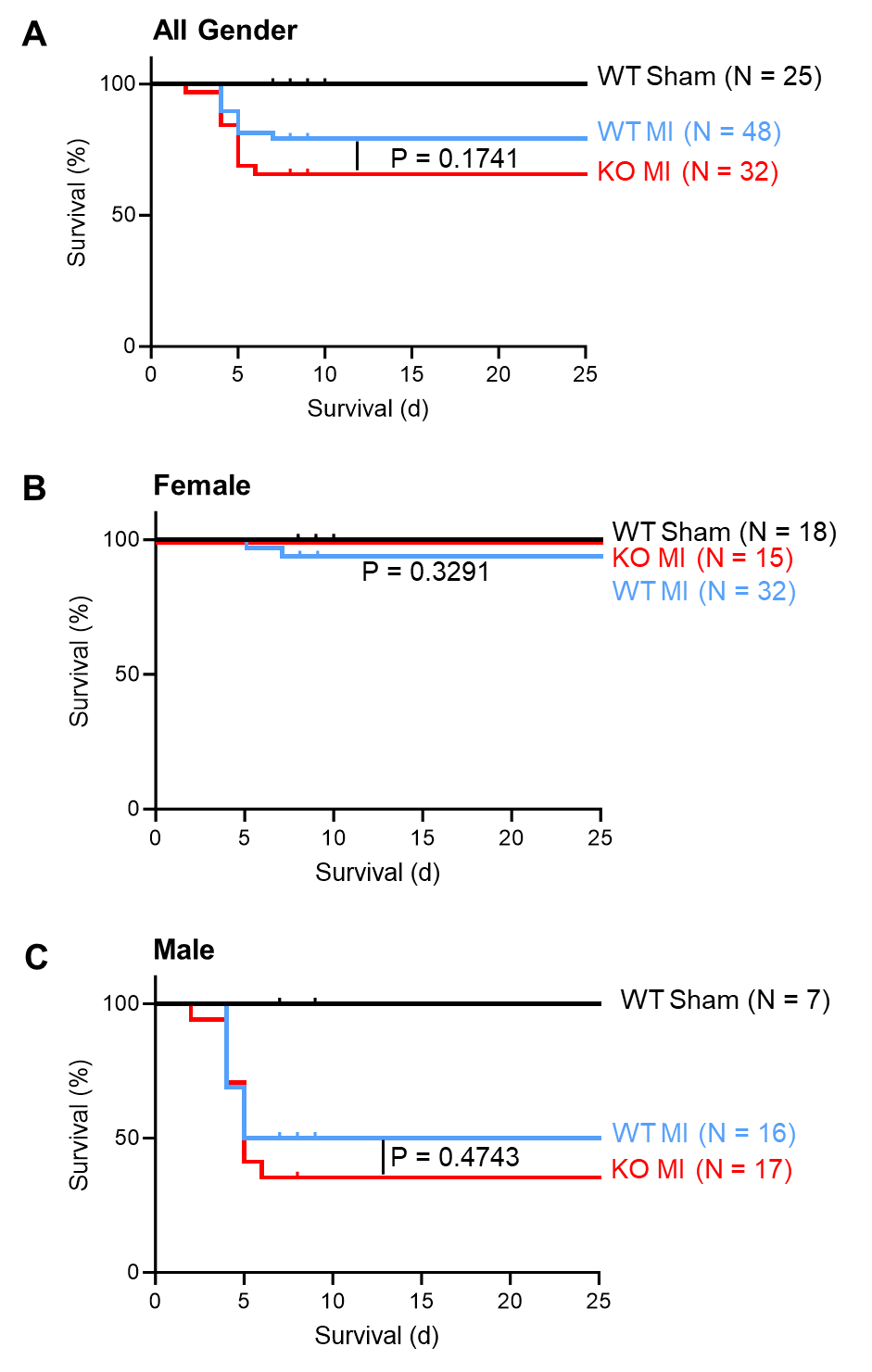


# Supplementary Figure 4. Survival analysis of wild-type and dysferlin-knockout mice after myocardial infarction.

**A** through **C**, Kaplan-Meier survival curves up to 25 days for wild-type (WT) and dysferlin-knockout (KO) mice after myocardial infarction (MI) and WT mice after sham operation. *P* values were calculated using the Log-rank (Mantel-Cox) test to compare WT vs KO mice after MI.


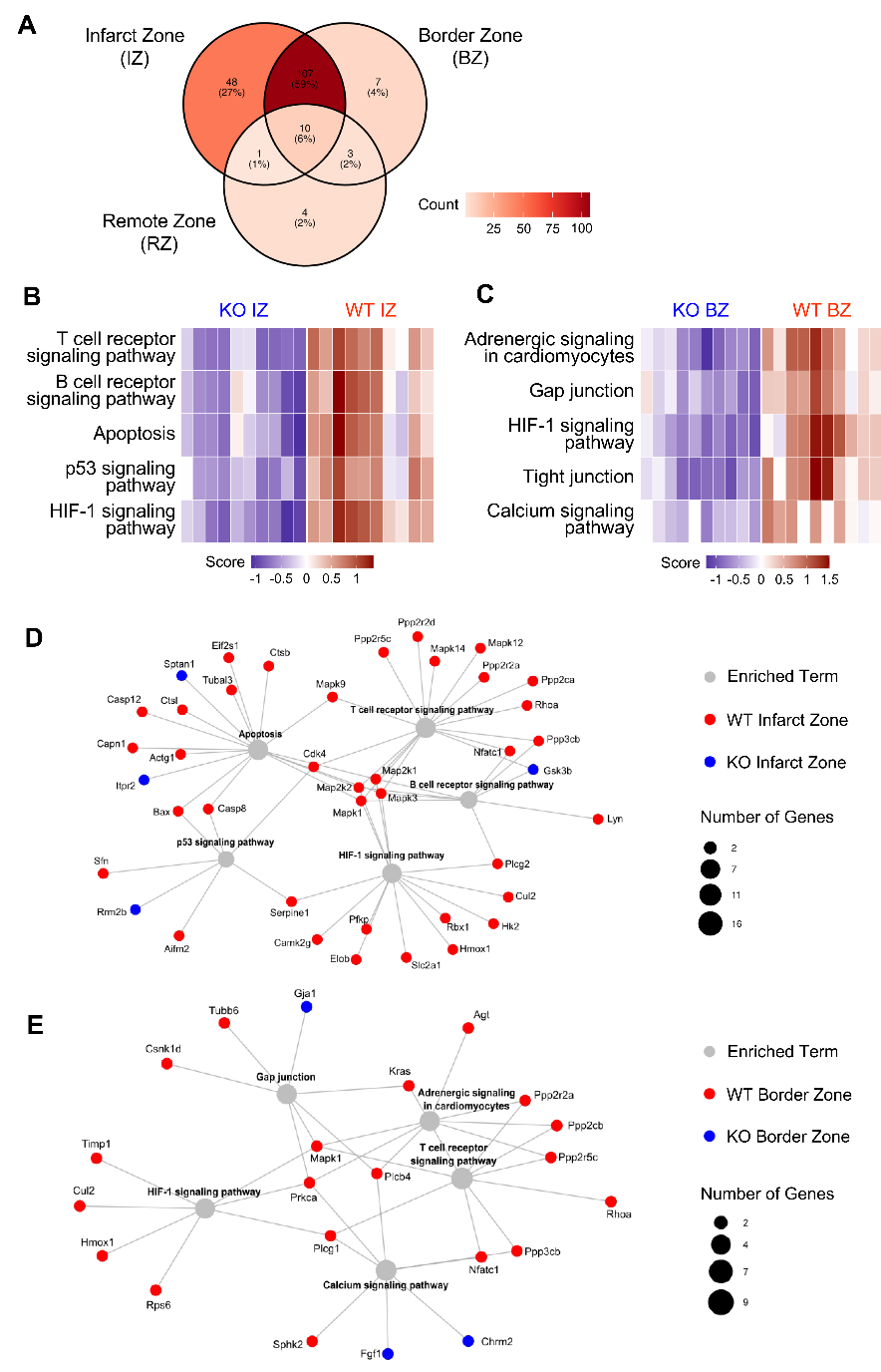


# Supplementary Figure 5. Comparison of enriched KEGG pathways in wild-type vs. dysferlin-knockout MI zone comparisons.

Differentially abundant proteins from the WT vs. dysferlin-knockout (KO) comparisons post-MI in Figure 2 were subjected to KEGG pathway analysis using pathfindR. *n*=5 different mouse hearts independently analysed per group with two replicate injections per sample. **A**, Venn diagram showing the overlap of enriched pathways across infarct zone (IZ), border zone (BZ), and remote zone (RZ) comparisons in WT vs. KO. **B** through **C**, Heatmaps highlighting 5 enriched KEGG pathways each from infarct zone (**B**) and border zone pairwise (**C**) comparisons. **D** through **E**, Term-gene graphs of the enriched KEGG pathways shown in B-C. Term circle size indicates the number of differentially abundant proteins linked to each KEGG pathway. Colours of the smaller gene circles point out up- or downregulation (blue, upregulation in KO/downregulation in WT; red, downregulation in KO/upregulation in WT).


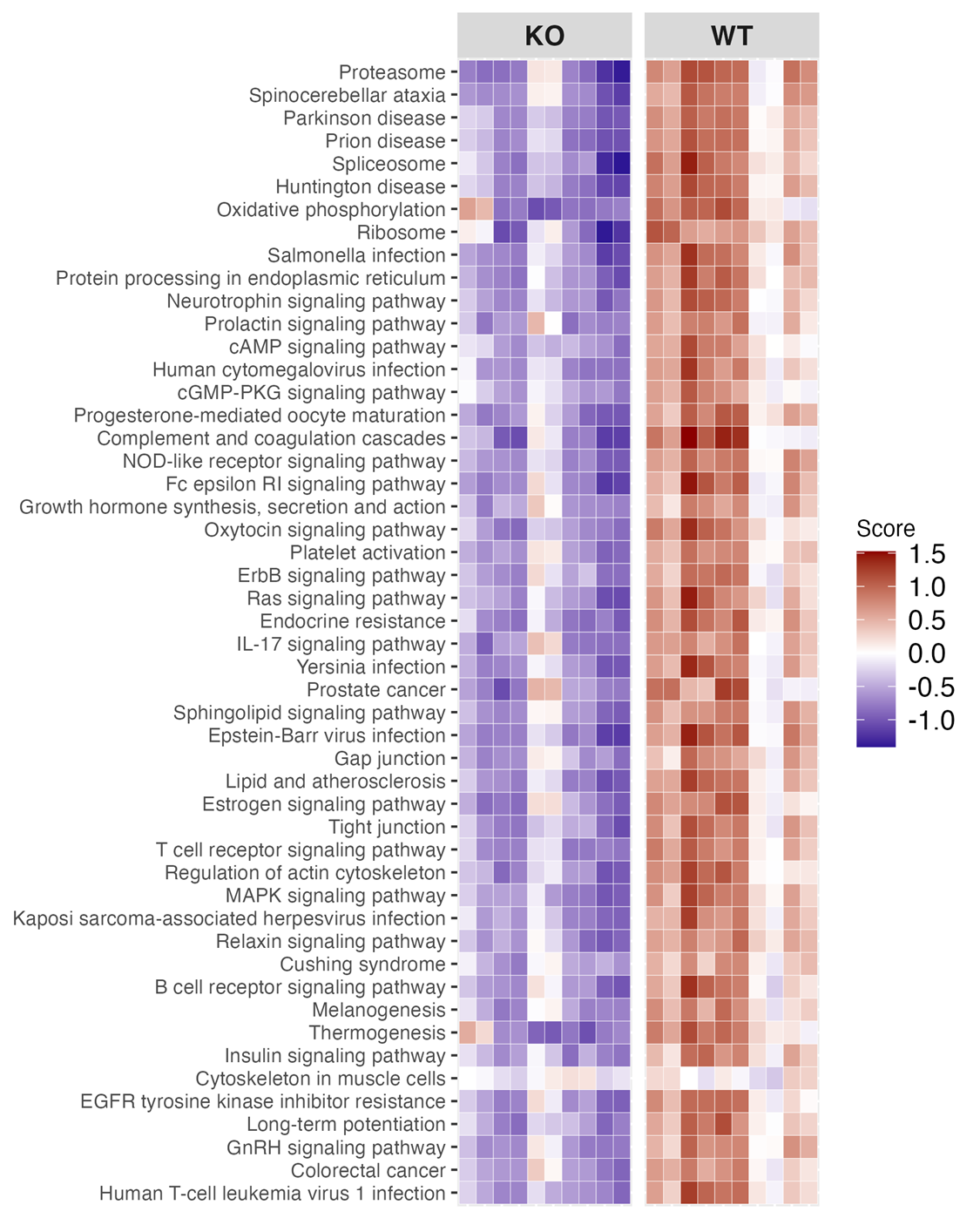


# Supplementary Figure 6. PathfindR analysis of KEGG pathways in the left-ventricular infarct zone of wild-type and dysferlin-knockout mice.

Top 50 enriched KEGG pathways, ranked by *P* value (most significantly enriched pathway at the top), comparing wild-type (WT) and dysferlin-knockout (KO) infarct zones, using PathfindR analysis. The heatmap shows pathway enrichment scores: Each row represents a KEGG pathway, and the blue-to-red colour scale reflects the relative enrichment between WT and KO samples. *n*=5 mice per group with 2 technical replicates each.


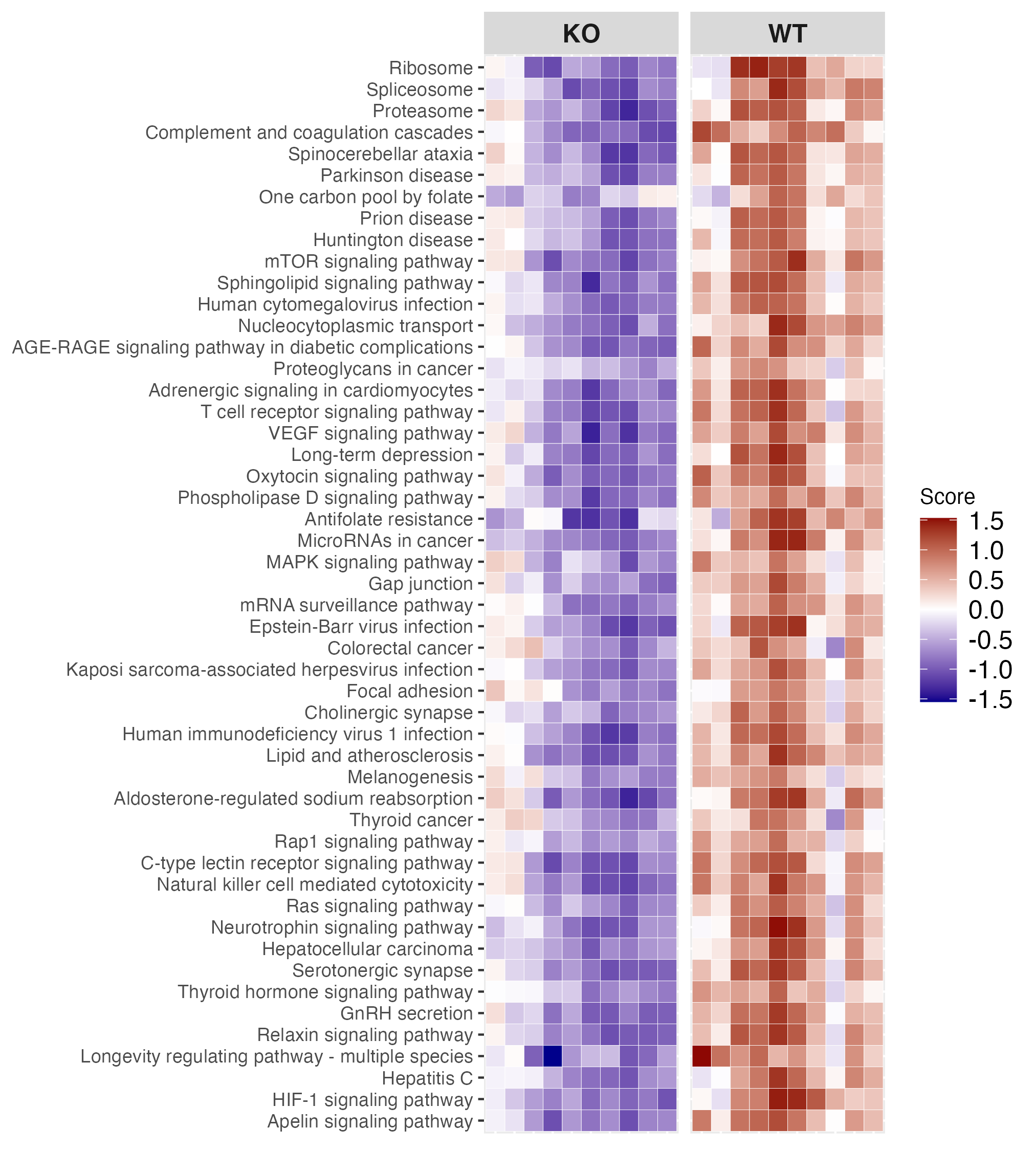


# Supplementary Figure 7. PathfindR analysis of KEGG pathways in the left-ventricular border zone of wild-type and dysferlin-knockout mice.

Top 50 enriched KEGG pathways, ranked by *P* value (most significantly enriched pathway at the top), comparing wild-type (WT) and dysferlin-knockout (KO) border zones, using PathfindR analysis. The heatmap shows pathway enrichment scores: Each row represents a KEGG pathway, and the blue-to-red colour scale reflects the relative enrichment between WT and KO samples. *n*=5 mice per group with 2 technical replicates each.


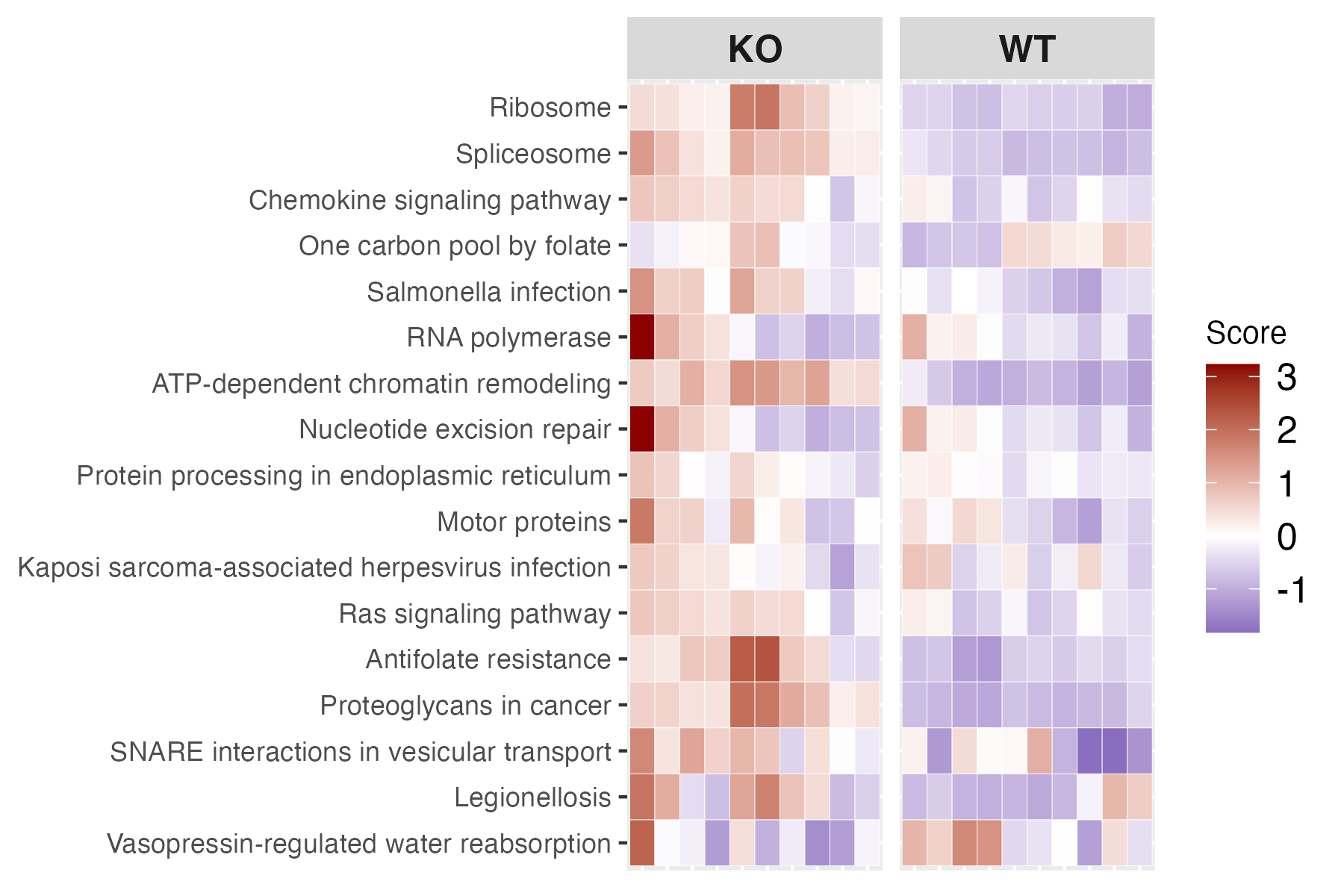


# Supplementary Figure 8. PathfindR analysis of KEGG pathways in the left-ventricular remote zone of wild-type and dysferlin-knockout mice.

Enriched KEGG pathways, ranked by *P* value (most significantly enriched pathway at the top), comparing wild-type (WT) and dysferlin-knockout (KO) remotes zones, using PathfindR analysis. The heatmap shows pathway enrichment scores: Each row represents a KEGG pathway, and the blue-to-red colour scale reflects the relative enrichment between WT and KO samples. *n*=5 mice per group with 2 technical replicates each.


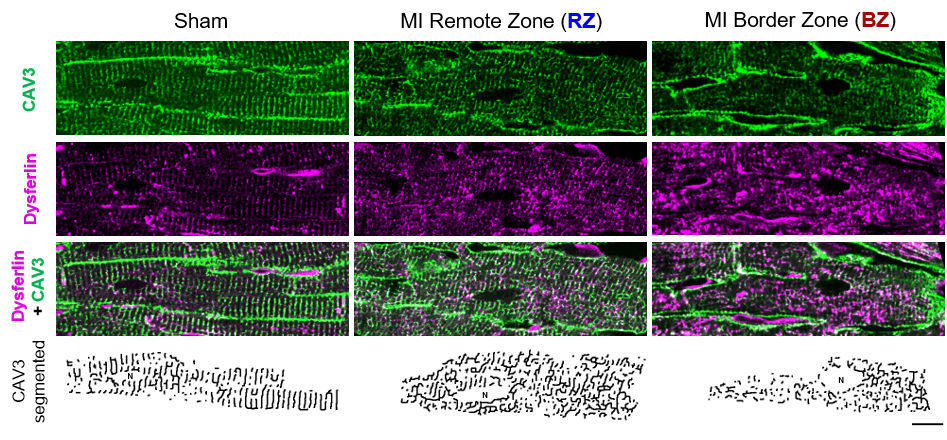


# Supplementary Figure 9. Representative confocal co-immunofluorescence images of dysferlin and caveolin-3 in left-ventricular tissue sections from wild-type mice post-MI.

Confocal co-immunofluorescence images of dysferlin (magenta) and the membrane and transverse-axial tubule (TAT) marker caveolin-3 (CAV3, green) in LV tissue slices from WT sham and myocardial infarction (MI) hearts 1-week post-surgery. Supplementary Figure 9 presents single-channel images corresponding to main Figure 3. Scale bar 10 µm.


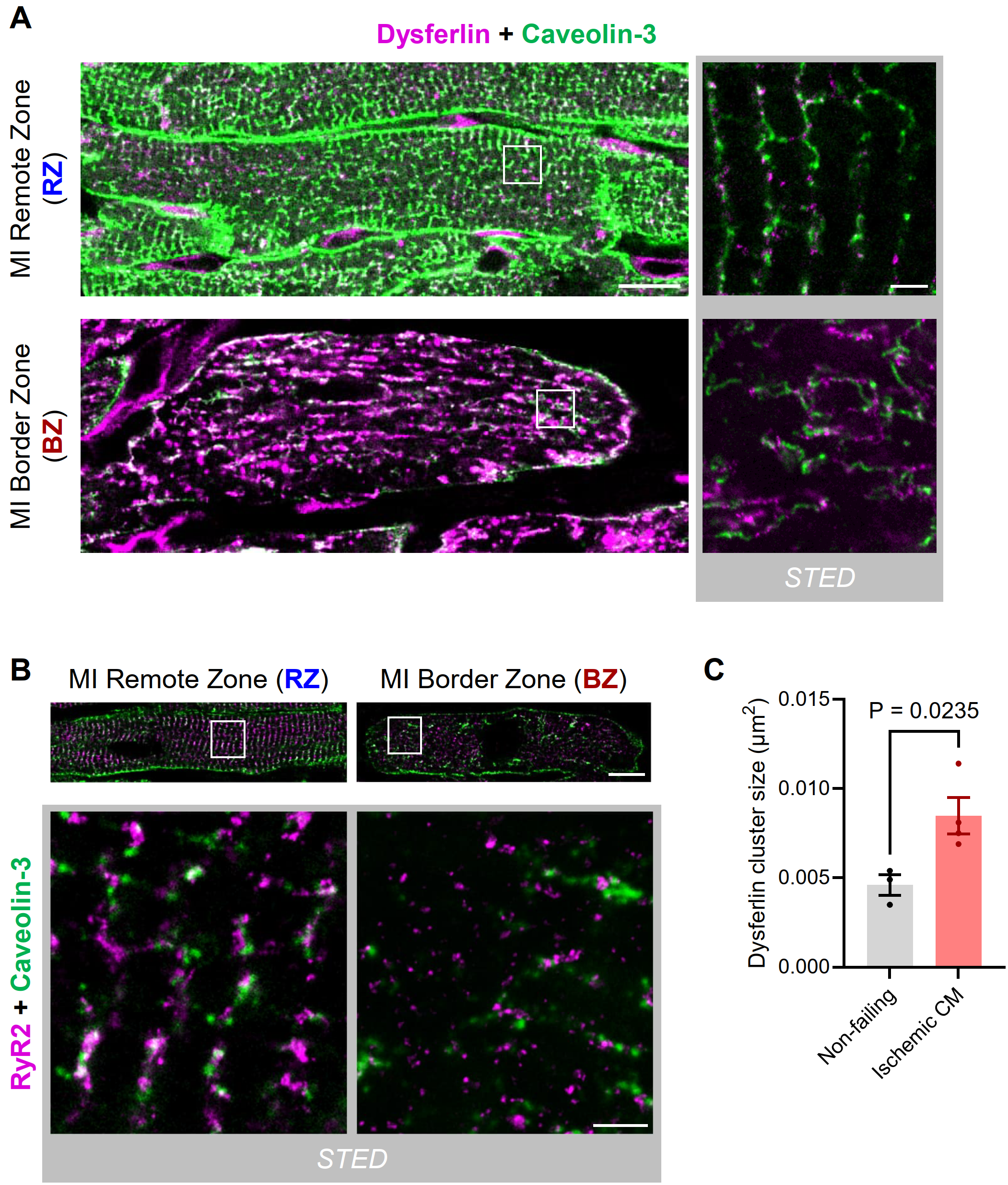


# Supplementary Figure 10. Dysferlin accumulations at residual TAT structures in cardiomyocytes of the MI border zone.

**A**, Representative confocal (left) and stimulated emission depletion (STED, right) co-immunofluorescence images of dysferlin (magenta) and the membrane and transverse-axial tubule (TAT) marker caveolin-3 (CAV3, green) in WT myocytes of the remote (RZ) and border zone (BZ) 1-week post-MI. White boxes highlight regions magnified on the right. Scale bar 10 µm (cell overviews), and 1µm (magnifications). **B**, Confocal (top) and stimulated emission depletion (STED, bottom) co-immunofluorescence images of RyR2 (magenta) and the membrane and transverse-axial tubule (TAT) marker caveolin-3 (CAV3, green) in WT myocytes of the remote (RZ) and border zone (BZ) 1-week post-MI. White boxes highlight regions magnified below. Scale bar 10 µm (cell overviews), and 1µm (magnifications). C, Dysferlin cluster size quantification in myocytes of LV tissue from human non-failing donor hearts and patients presented with ischemic cardiomyopathy (CM), corresponding to Figure 4L. *n*=3 non-failing vs 3 ischemic CM patients. Unpaired Welch *t* test.


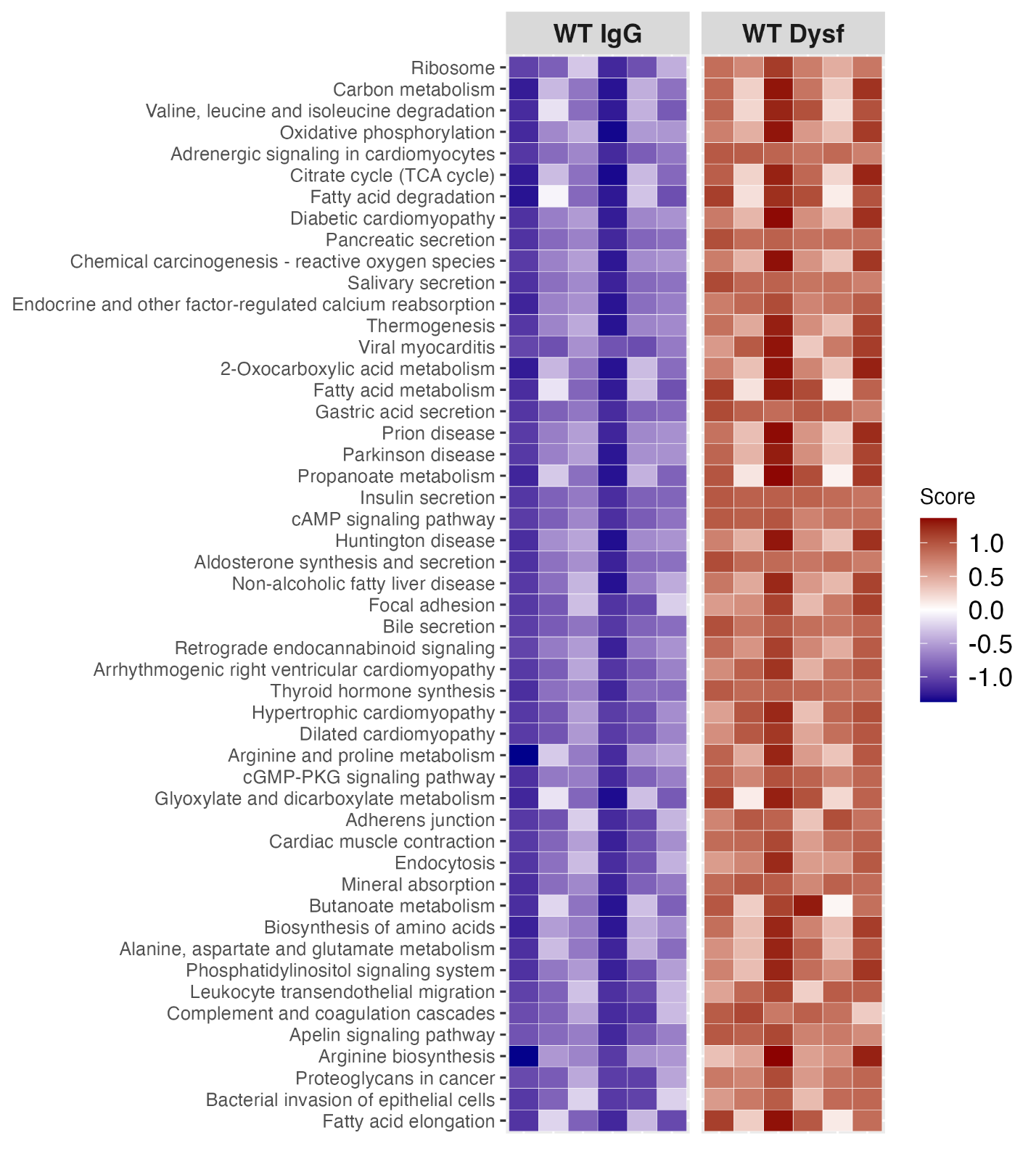


# Supplementary Figure 11. PathfindR analysis of KEGG pathways of the anti-dysferlin co-immunoprecipitation from left-ventricular myocardial lysates compared with unspecific IgG control.

Top 50 out of 135 enriched KEGG pathways, ranked by *P* value (most significantly enriched pathway at the top), comparing anti-dysferlin co-immunoprecipitation from wild-type (WT) LV myocardial lysates with unspecific IgG control, using PathfindR analysis. The heatmap shows pathway enrichment scores: Each row represents a KEGG pathway, and the blue-to-red colour scale reflects the relative enrichment between anti-dysferlin and unspecific IgG. *n*=3 mice per group with 2 technical replicates each.


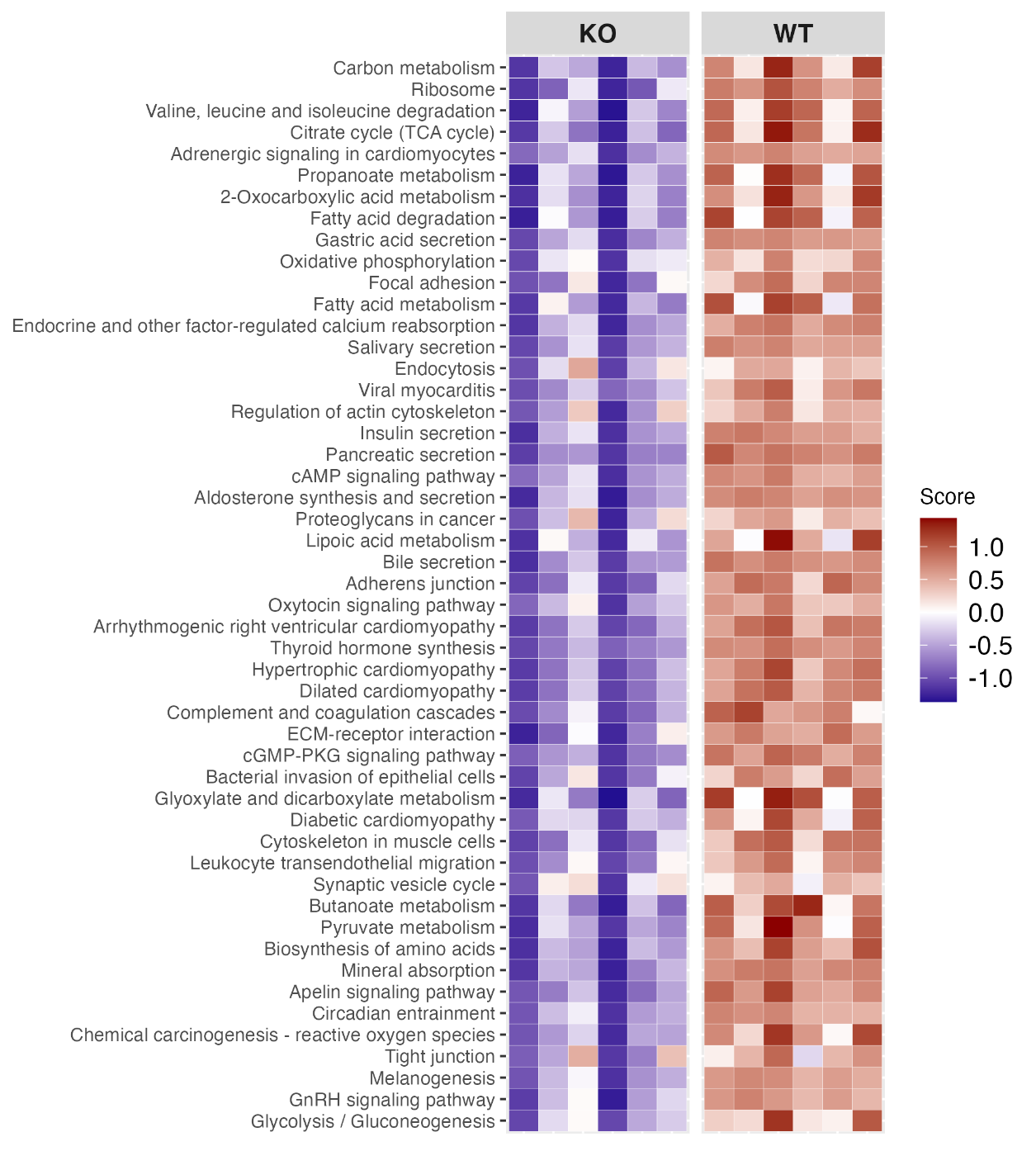


# Supplementary Figure 12. PathfindR analysis of KEGG pathways of the anti-dysferlin co-immunoprecipitation from wildtype vs. dysferlin-knockout left-ventricular myocardial lysates.

Top 50 out of 145 enriched KEGG pathways, ranked by *P* value (most significantly enriched pathway at the top), comparing anti-dysferlin co-immunoprecipitation from wild-type (WT) vs dysferlin-knockout (KO) LV myocardial lysates, using PathfindR analysis. The heatmap shows pathway enrichment scores: Each row represents a KEGG pathway, and the blue-to-red colour scale reflects the relative enrichment between WT and KO. *n*=3 mice per group with 2 technical replicates each.


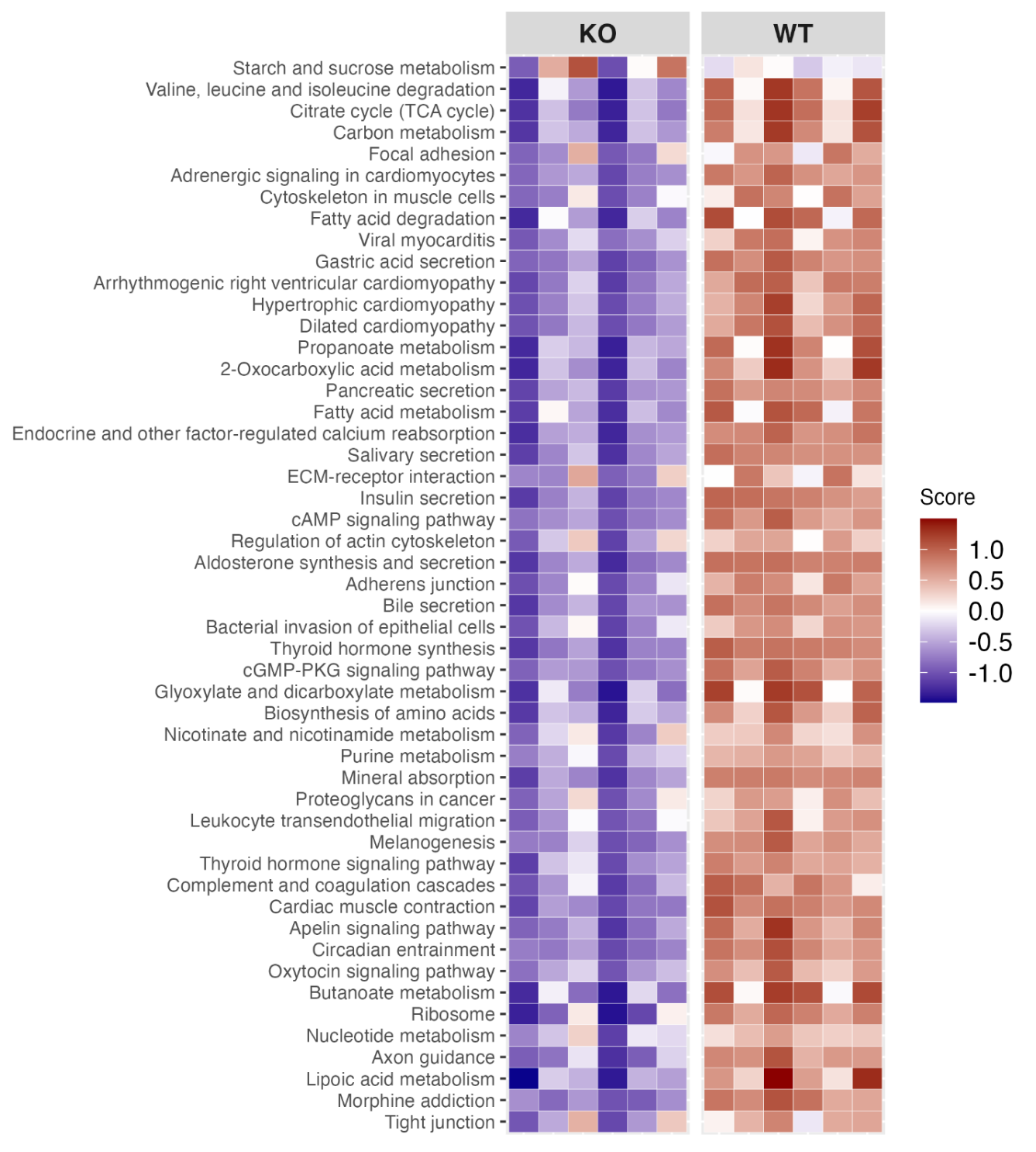


# Supplementary Figure 13. PathfindR analysis of KEGG pathways of the anti-dysferlin co-immunoprecipitation from wildtype vs. dysferlin-knockout left-ventricular myocardial lysates in presence of 1 mM [Ca^2+^].

Top 50 out of 139 enriched KEGG pathways, ranked by *P* value (most significantly enriched pathway at the top), comparing anti-dysferlin co-immunoprecipitation from wild-type (WT) vs dysferlin-knockout (KO) LV myocardial lysates in presence of 1 mM [Ca^2+^], using PathfindR analysis. The heatmap shows pathway enrichment scores: Each row represents a KEGG pathway, and the blue-to-red colour scale reflects the relative enrichment between WT and KO. *n*=3 mice per group with 2 technical replicates each.


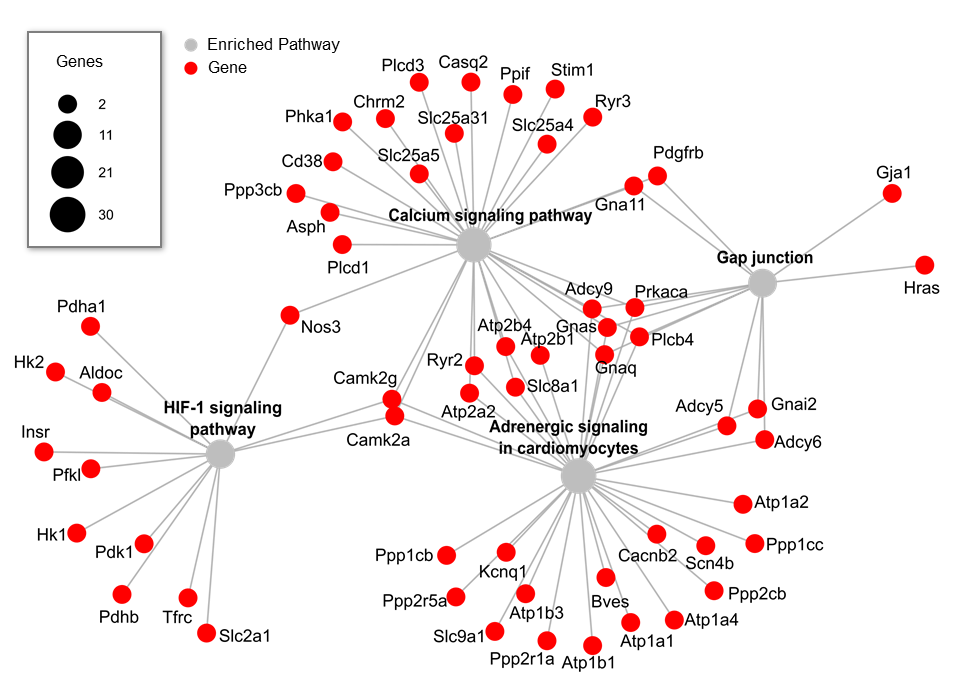


# Supplementary Figure 14. PathfindR term-gene graph of KEGG pathways of the anti-dysferlin co-immunoprecipitation from wildtype vs. dysferlin-knockout left-ventricular myocardial lysates.

Highlighted are 4 out of 145 enriched KEGG pathways. Term circle size indicates the number of differentially abundant proteins linked to each KEGG pathway. *n*=3 mice per group with 2 technical replicates each.


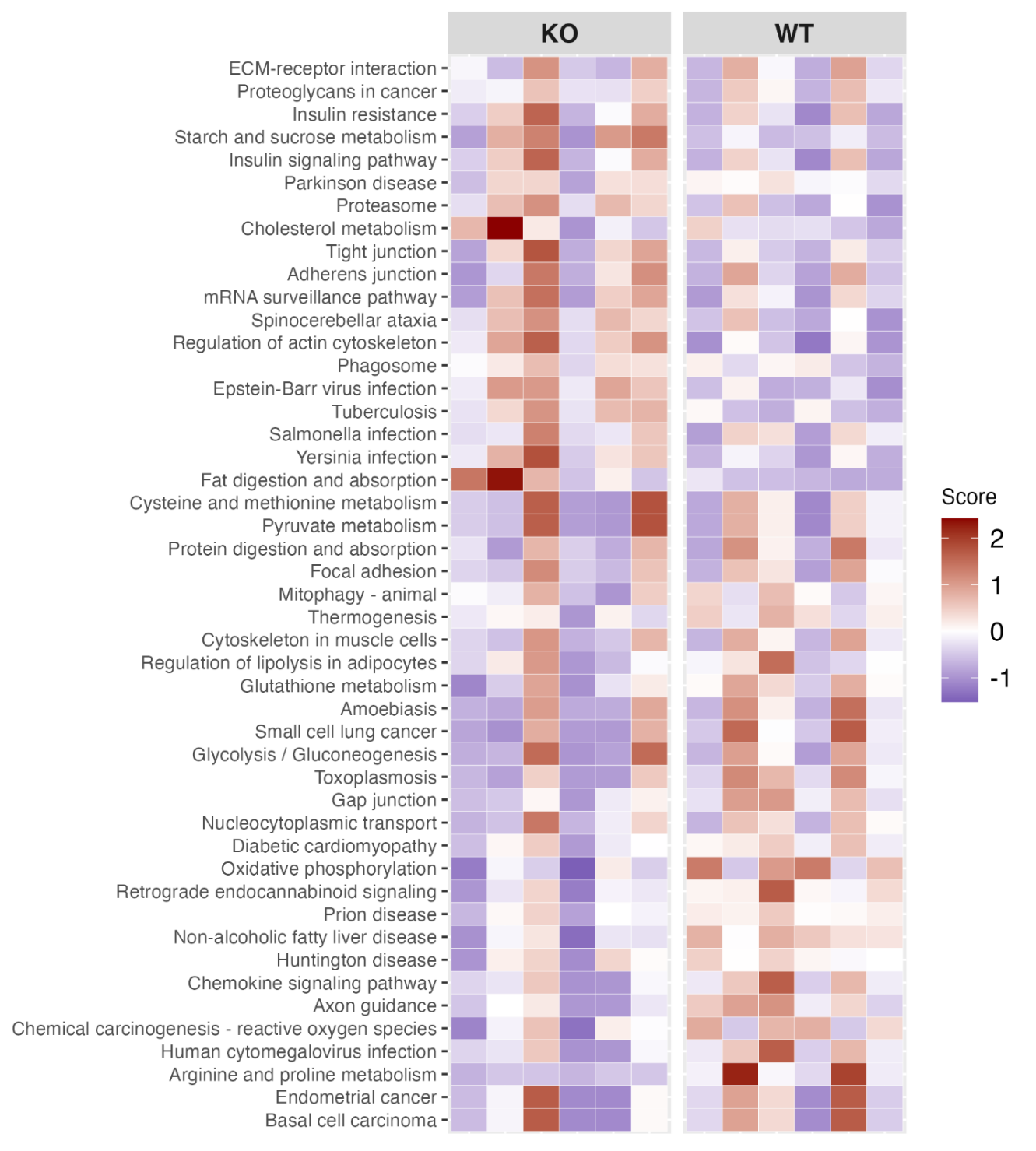


# Supplementary Figure 15. PathfindR analysis of KEGG pathways from proteins only enriched in presence of 1 mM [Ca^2+^] in the anti-dysferlin co-immunoprecipitation from wildtype vs. dysferlin-knockout left-ventricular myocardial lysates.

Top 47 enriched KEGG pathways from proteins only enriched in presence of 1 mM [Ca^2+^] in the anti-dysferlin co-immunoprecipitation from wildtype (WT) vs. dysferlin-knockout (KO) LV myocardial lysates (not in absence of calcium), ranked by *P* value (most significantly enriched pathway at the top), using PathfindR analysis. The heatmap shows pathway enrichment scores: Each row represents a KEGG pathway, and the blue-to-red colour scale reflects the relative enrichment between WT and KO. *n*=3 mice per group with 2 technical replicates each.

# Supplementary Figure 16. Cluster density of dysferlin and connexin-43 at the intercalated disc membrane folds.

Detailed dysferlin and connexin-43 (CX43) cluster analysis in manually selected ROIs of ICD membrane folds in WT LV myocytes identifies a substantially higher dysferlin than connexin-43 cluster density. Supplementary Figure 16 corresponds to main Figure 6. *n*=48 LV myocytes of 5 individual WT hearts. Paired *t* test.


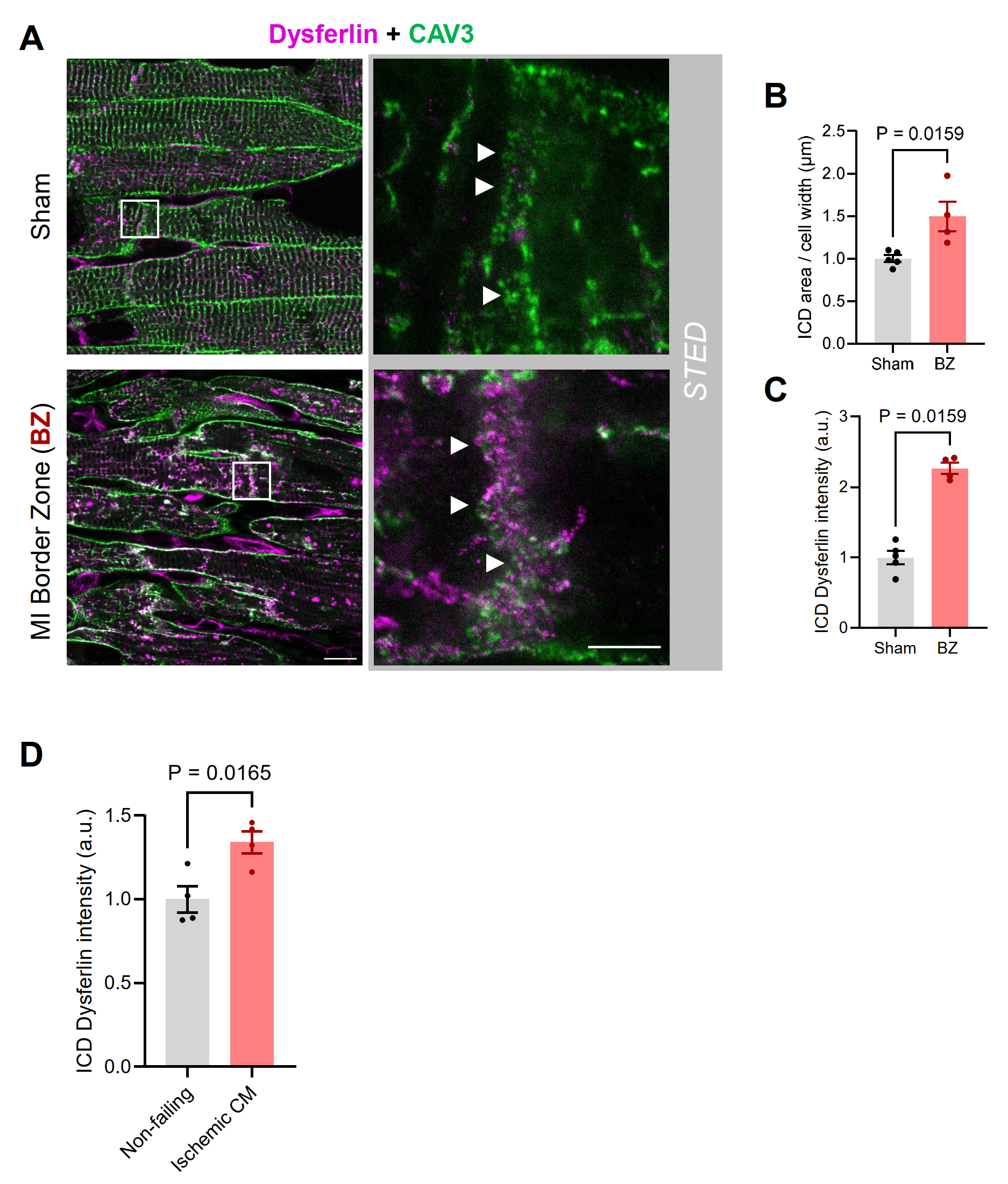


# Supplementary Figure 17. STED co-immunofluorescence imaging of dysferlin and Caveolin-3 at the intercalated discs in cardiomyocytes of the MI border zone.

**A**, Confocal (left) and STED (right) co-immunofluorescence imaging of dysferlin (magenta) and the membrane marker caveolin-3 (green) at the intercalated disc (ICD) membrane folds (arrowheads) in LV tissue slices from WT sham hearts and the myocardial infarction (MI) border zone 1-week post-surgery. Scale bars 10 µm (overviews) and 2 µm (magnifications). White boxes indicate magnified regions on the right. **B** through **C**, The ICD area normalized by local cell width and the dysferlin signal intensity are significantly increased in cardiomyocytes of the MI border zone compared to myocytes of sham-treated hearts. *n*=24/20 LV myocytes of 5 sham controls and 4 BZ of individual mouse hearts in **A**, *n*=27/24 LV myocytes of 5 sham controls and 4 BZ of individual mouse hearts in **B**. Mann Whitney *U* test. **D**, Dysferlin signal intensity quantification at the ICD of cardiomyocytes in LV tissue from human non-failing donor hearts and patients presented with ischemic cardiomyopathy (CM), corresponding to Figure 7J. *n*=4 non-failing vs 4 ischemic CM patients. Unpaired Welch *t* test.


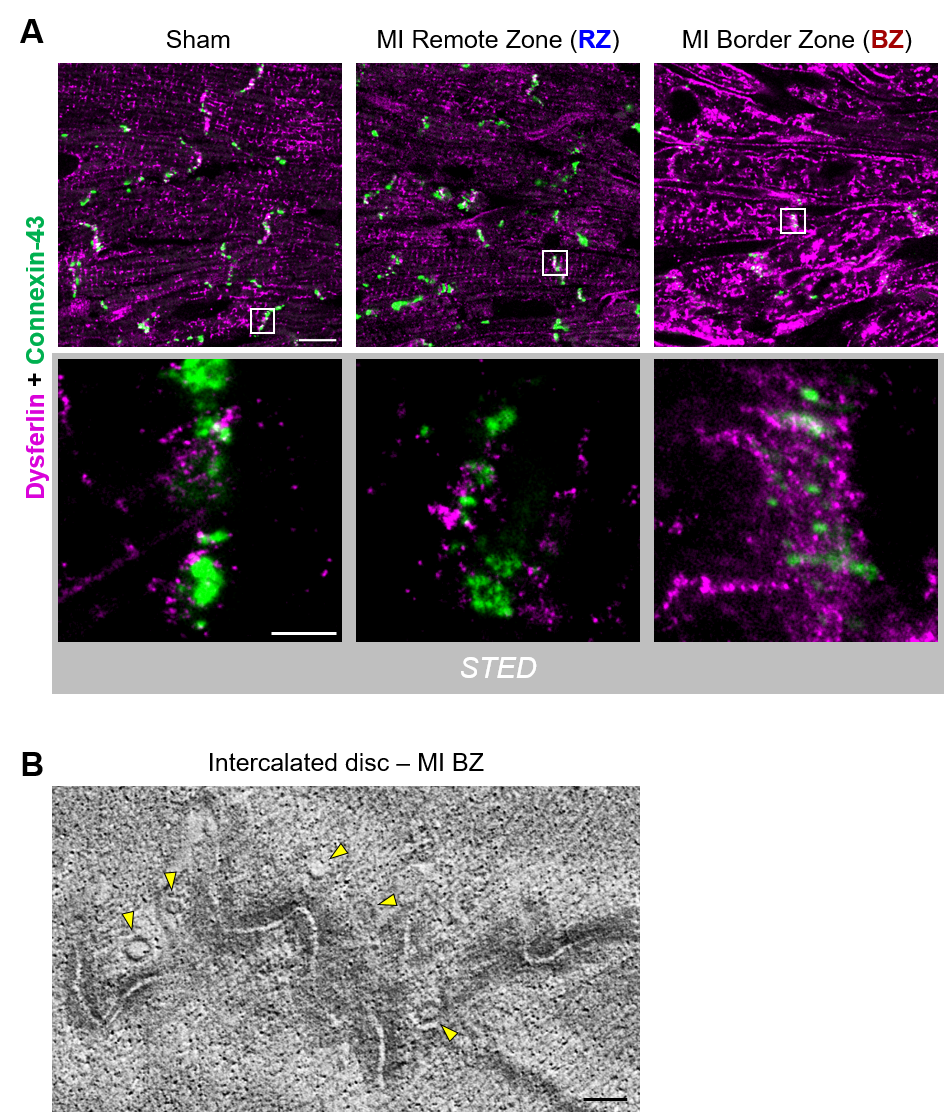


# Supplementary Figure 18. STED co-immunofluorescence imaging of dysferlin and connexin-43 at the intercalated discs in cardiomyocytes of the MI remote and border zone.

**A**, Confocal (top) and STED (bottom) co-immunofluorescence imaging of dysferlin and the gap junctions protein connexin-43 (CX43) at the intercalated disc (ICD) cell-cell contact sites of LV myocytes in sham hearts, and in the remote vs border zone of WT mice 1-week post-MI. Scale bars 10 µm (top) and 1 µm (bottom). White boxes indicate magnified regions below. **B**, Electron tomography confirms numerous intracellular vesicles (yellow arrowheads) in close proximity to the ICD membrane folds in the MI border zone. Scale bar 200 nm.

# Supplementary Table 1. Primary antibodies used in immunoblotting and immunofluorescence imaging as referenced in the figure legends or methods section.

| **Method** | **Buffer** | **Composition** |
| --- | --- | --- |
| Immunohistology | Permeabilisation buffer | 4% BSA in PBS (pH 7.4), 0,1% Triton X-100 |
|  | Blocking buffer | 4% BSA in PBS (pH 7.4) |
| CoIP | Lysis buffer | 150 NaCl, 50 Tris/HCl, 1 EDTA, 0.15% (w/v) CHAPS, pH 7.4 |
|  | Lysis buffer with Ca^2+^ | 150 NaCl, 50 Tris/HCl, 1 EDTA, 2 CaCl_2_, 0.15% (w/v) CHAPS, pH 7.4 |
| Western Blot | Homogenization buffer | 10 HEPES, 300 sucrose, 150 NaCl, 1 EGTA, 2 CaCl_2_, 0.5% (v/v) Triton X-100, protease and phosphatase inhibitor mix (Roche), pH 7.4 |
|  | Running buffer | 24.93 Tris-base, 191.82 Glycine, 3.47 SDS |
|  | Transfer buffer | 24.93 Tris-base, 191.82 Glycine, 20 % (v/v) Methanol |
|  | TBST | 170 NaCl, 9.99 Tris-Base, 0.001% (v/v) Tween 20, pH 7.5 |
|  | Blocking Buffer | 5% (w/v) non-fat milk powder in TBST |
| DIA-MS | SDS lysis buffer | 2% SDS, 100 mM HEPES, protease and phosphatase inhibitor mix (Roche), pH 8.0 |

# Supplementary Table 2. Composition of buffer solutions as referenced in the methods section in mmol/L.

# Supplementary Table 3. Left ventricular mouse echocardiography 1 week post-MI.

Means ± SEM. AWth, anterior wall thickness; LVEDD, left-ventricular end-diastolic diameter; LVESD, left-ventricular end-systolic diameter; PWTh, posterior wall thickness, FS, fractional shortening; FAS; fractional area shortening; EF, ejection fraction. Ns, not significant; sig, significant.

# References

1. Bansal D, Miyake K, Vogel SS, Groh S, Chen CC, Williamson R, McNeil PL, Campbell KP. Defective membrane repair in dysferlin-deficient muscular dystrophy. *Nature* 2003;**423**:168-172.

2. Gu Z, Eils R, Schlesner M. Complex heatmaps reveal patterns and correlations in multidimensional genomic data. *Bioinformatics* 2016;**32**:2847-2849.

3. Wickham H, Averick M, Bryan J, Chang W, D'Agostino McGowan L, Francois R, Grolemund G, Hayes A, Henry L, Hester J, Kuhn M, Pedersen TL, Miller E, Bache SM, Müller K, Ooms J, Robinson D, Seidel DP, Spinu V, Takahashi K, Vaughan D, Wilke C, Woo K, Yutani H. Welcome to the Tidyverse. *J Open Source Softw* 2019;**4**:1686.

4. Yu G, Wang LG, Han Y, He QY. clusterProfiler: an R package for comparing biological themes among gene clusters. *OMICS* 2012;**16**:284-287.

5. Ulgen E, Ozisik O, Sezerman OU. pathfindR: An R Package for Comprehensive Identification of Enriched Pathways in Omics Data Through Active Subnetworks. *Front Genet* 2019;**10**:858.

6. Alsina KM, Hulsurkar M, Brandenburg S, Kownatzki-Danger D, Lenz C, Urlaub H, Abu-Taha I, Kamler M, Chiang DY, Lahiri SK, Reynolds JO, Quick AP, Scott L, Jr., Word TA, Gelves MD, Heck AJR, Li N, Dobrev D, Lehnart SE, Wehrens XHT. Loss of Protein Phosphatase 1 Regulatory Subunit PPP1R3A Promotes Atrial Fibrillation. *Circulation* 2019;**140**:681-693.

7. Brandenburg S, Pawlowitz J, Eikenbusch B, Peper J, Kohl T, Mitronova GY, Sossalla S, Hasenfuss G, Wehrens XH, Kohl P, Rog-Zielinska EA, Lehnart SE. Junctophilin-2 expression rescues atrial dysfunction through polyadic junctional membrane complex biogenesis. *JCI Insight* 2019;**4**.

8. Rog-Zielinska EA, Scardigli M, Peyronnet R, Zgierski-Johnston CM, Greiner J, Madl J, O'Toole ET, Morphew M, Hoenger A, Sacconi L, Kohl P. Beat-by-Beat Cardiomyocyte T-Tubule Deformation Drives Tubular Content Exchange. *Circ Res* 2021;**128**:203-215.
